# Hybrid tungstate-functionalized nitrogen-doped rice husk biochar for selective dual adsorption of methylene blue and methyl orange

**DOI:** 10.1039/d6ra02755e

**Published:** 2026-08-05

**Authors:** Walid M. Youssef, Entsar H. Taha, Adel A. El-Zahhar, Majed M. Alghamdi, Mohamed H. Taha

**Affiliations:** a Nuclear Materials Authority P.O. Box 530, El Maddi Cairo Egypt m.walid_nma@yahoo.com; b Department of Plant Protection, Faculty of Agriculture, Ain Shams University Cairo Egypt; c Department of Chemistry, Faculty of Science, King Khalid University P.O.Box 9004 Abha 61413 Saudi Arabia

## Abstract

The development of multifunctional and sustainable sorbents for simultaneous removal of oppositely charged dyes remains a significant challenge in wastewater treatment. In this study, a tungstate-modified rice husk biochar composite (WRHB) was engineered through the synergistic integration of K_2_CO_3_ activation, nitrogen heteroatom doping, and sodium tungstate functionalization. The resulting hybrid material exhibits a mesoporous structure (SBET = 145.4 m^2^ g^−1^; pore diameter ≈ 3.8 nm), an alkaline surface charge (pH_pzc_ ≈ 8.6), and uniformly dispersed crystalline Na_2_WO_4_ domains within an amorphous carbon matrix. Adsorption behavior toward methylene blue (MB) and methyl orange (MO) was systematically investigated under identical conditions. Under equilibrium isotherm conditions of pH 6.01, an initial dye concentration range of 20–200 mg L^−1^, a WRHB dosage of 3.0 g L^−1^, a contact time of 240 min, and a temperature of 25 ± 1 °C, maximum Langmuir monolayer capacities reached 24.2 mg g^−1^ for MB and 31.5 mg g^−1^ for MO, with Sips modeling confirming quasi-monolayer adsorption on energetically comparable sites. Kinetic data followed the pseudo-second-order model, indicating surface-site-controlled uptake with multi-step diffusion contributions. Thermodynamic analysis revealed spontaneous and exothermic adsorption (Δ*G*° < 0; Δ*H*° = −23.4 and −46.3 kJ mol^−1^ for MB and MO, respectively), with stronger affinity toward MO. Mechanistically, adsorption proceeds through cooperative electrostatic attraction, π–π stacking, hydrogen bonding, and ion–dipole interactions involving tungstate-derived polar domains. The sorbent demonstrated high regenerability using dilute mineral acids and maintained strong decolorization efficiency (≈85%) in real textile wastewater. The combined structural tunability, dual-dye functionality, and operational stability position WRHB as a scalable and application-oriented platform for dye-contaminated wastewater remediation.

## Abstract 2

Tungstate-functionalized nitrogen-doped rice husk biochar enables dual adsorption of cationic methylene blue and anionic methyl orange, with efficient acid-assisted desorption and strong decolorization of real textile wastewater.

## Graphical abstract

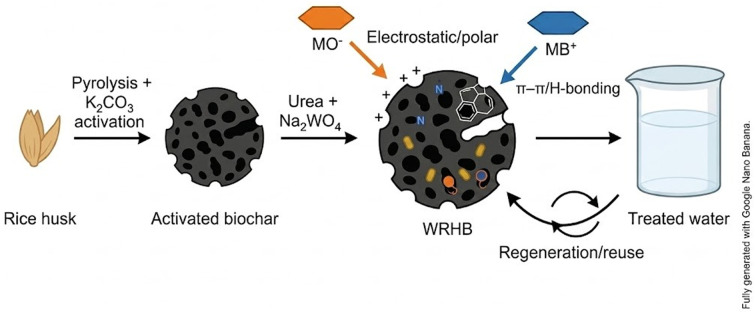

## 1 Introduction

Industrial wastewater contaminated with synthetic dyes remains a persistent environmental concern due to the high chemical stability, strong coloration, and resistance of many dyes to natural degradation processes. Effluents from textile dyeing, leather processing, printing, and paper manufacturing frequently contain complex mixtures of dyes that adversely affect water quality even at low concentrations. Beyond aesthetic deterioration, dye contamination reduces light penetration, disrupts aquatic ecosystems, and may introduce toxic or mutagenic species, emphasizing the need for effective and sustainable treatment strategies.^1–3^ Methylene blue (MB) and methyl orange (MO) are widely used as representative cationic and anionic dyes, respectively, owing to their contrasting molecular structures, charge characteristics, and environmental behavior. Their coexistence in wastewater streams increases treatment complexity, as adsorption efficiency and selectivity are strongly governed by surface chemistry, electrostatic interactions, and solution conditions. Consequently, developing sorbents capable of efficiently capturing both dye types while maintaining stability and reusability remains a central objective in adsorption-based wastewater treatment research.^2–4^

A variety of physicochemical and biological techniques—including coagulation–flocculation,^5^ membrane separation,^6^ advanced electrochemical loxidation processes,^7^ and biodegradation^8^—have been investigated for dye removal. However, many of these approaches are constrained by high energy or chemical consumption, incomplete removal of recalcitrant dyes, secondary waste generation, or reduced efficiency in complex effluent matrices.^9–11^ In contrast, adsorption remains a versatile and operationally simple method due to its high removal efficiency, adaptability, and compatibility with existing treatment systems. Its large-scale applicability, however, is closely linked to the availability of low-cost, regenerable, and environmentally benign adsorbent materials.^11–13^

Carbon-based adsorbents remain key players in dye removal due to their adaptable pore structure and strong attraction to organic pollutants. While commercial activated carbons have excellent adsorption capabilities, their high production cost and reliance on non-renewable raw materials hinder the economic viability for large-scale usage.^2,3^ Growing awareness of these limitations has led to a rising interest in carbons and biochars derived from agricultural residues, which provide distinct benefits in waste utilization, cost minimization, and environmental sustainability.^14–16^ Rice husk stands out among different feedstocks thanks to its widespread availability and distinct composition, which comprises a carbon-rich matrix along with a high inherent silica content. Its dual carbon–silica composition offers potential for customizing pore structure and surface properties.^15,17^ Rice-husk-derived biochars synthesized under low-pressure conditions typically show restricted surface area, inadequate pore accessibility, and insufficient surface reactivity, leading to moderate adsorption capacities for organic dyes. Therefore, targeted activation and surface modification strategies are needed to improve their adsorption performance.^2,3^

Recent critical reviews of biochar-based, graphene-based, and other sustainable carbon materials indicate that pollutant adsorption is governed by the coupled effects of pore architecture, surface charge, functional groups, and solution conditions, while pore engineering, heteroatom doping, surface modification, and inorganic–carbon composite formation provide complementary routes for improving adsorption-site accessibility, selectivity, stability, and reusability.^18–20^ Alkali activation (*e.g.*, KOH, K_2_CO_3_, Na_2_CO_3_) is widely used to promote pore development and generate hierarchical micro–mesoporous structures, thereby improving mass transfer and adsorbate accessibility.^21–23^ In parallel, heteroatom doping—particularly nitrogen incorporation—introduces electron-rich functional groups that enhance electrostatic attraction, π–π interactions, hydrogen bonding, and Lewis acid–base interactions with dye molecules.^23–26^ As a result, nitrogen-doped biochars frequently show improved adsorption performance toward both cationic and anionic dyes compared with undoped counterparts.^25–28^ Despite these advances, most reported systems employ either activation or heteroatom doping alone, and the synergistic integration of multiple modification strategies within a single rice-husk-derived sorbent remains insufficiently explored.

Consistent with these principles, evidence from binary and mixed-dye systems indicates that physically activated biochars can adsorb both methyl orange and methylene blue, yet adsorption behavior is often highly pH-dependent and divergent for oppositely charged dyes under identical conditions.^2^ Mixed-dye and dynamic investigations further reveal pronounced competitive adsorption and transport limitations, emphasizing the need for sorbents with accessible pore networks and stable surface functionality.^29,30^ In rice-derived materials, surface alkalinity and charge regulation play critical roles, as evidenced by rice husk ash and mineral-modified biochars that preferentially enhance cationic or anionic dye uptake through oxide-type surface sites.^31,32^ Building on this concept, metal-site engineering has emerged as an effective strategy: tungsten-coated biochars and tungstate-based biochar composites demonstrate enhanced methylene blue adsorption and strong interfacial interactions between tungsten oxide/tungstate domains and dye molecules, while the carbon matrix ensures adsorption capacity and structural stability.^33–35^ Although many of these systems incorporate photocatalysis, adsorption consistently governs overall pollutant removal efficiency.^34–37^ Despite these advances, rice-husk-derived and other biomass-based biochars commonly exhibit strong selectivity toward cationic dyes, whereas anionic dye adsorption remains limited or highly pH-dependent.^36,38^ Moreover, direct comparison of cationic and anionic dye uptake is often hindered by the use of different materials or experimental conditions.^2,29,30^ Importantly, the synergistic integration of alkali activation, nitrogen heteroatom doping, and tungstate-derived inorganic domains within a single rice-husk-derived biochar has not yet been systematically explored, particularly for dual-dye adsorption under identical conditions and performance validation in real wastewater matrices.^33–37^

The present study reports the development of a rice husk biochar hybrid composite engineered through the synergistic integration of alkali activation, nitrogen heteroatom functionalization, and tungstate-derived metal oxide incorporation. This multilevel design aims to generate a hierarchically porous structure with abundant electron-rich and oxygen-containing functional sites, enabling enhanced electrostatic and non-electrostatic interactions with both cationic and anionic dyes. The adsorption behavior toward methylene blue and methyl orange is systematically investigated under identical experimental conditions, including the effects of solution pH, contact time, sorbent dosage, initial dye concentration, and temperature. Kinetic, equilibrium isotherm, and thermodynamic analyses are employed to elucidate uptake behavior and feasibility, while regeneration experiments evaluate reusability using common mineral acids. Sorbent performance is further assessed in real wastewater to examine robustness under competitive conditions. By combining sustainable material design with comprehensive adsorption analysis and application-oriented validation, this work advances rice-husk-derived biochar composites as efficient, multifunctional sorbents for practical dye-contaminated wastewater treatment.

## 2 Experimental

### 2.1 Materials

Raw rice husk, used as the biomass precursor for biochar preparation, was collected from a local agricultural source and employed as received after thorough washing with deionized water to remove surface-adhered dust and water-soluble impurities. Potassium carbonate (K_2_CO_3_, analytical grade) was used as a chemical activating agent for biochar activation. Urea (analytical grade) served as the nitrogen precursor for heteroatom doping, while sodium tungstate (Na_2_WO_4_·*x*H_2_O, analytical grade) was utilized as the source of tungstate species for inorganic functionalization of the biochar matrix. All chemicals were purchased from commercial suppliers and used without further purification. Deionized water was used throughout all synthesis, washing, and impregnation procedures. Methylene blue (MB) and methyl orange (MO) dyes were selected as representative cationic and anionic adsorbates to evaluate the adsorption performance of the prepared sorbent. Analytical-grade MB (C_16_H_18_ClN_3_S·*x*H_2_O) and MO (C_14_H_14_N_3_NaO_3_S) were used to prepare stock dye solutions (1000 mg L^−1^), from which working solutions of the desired concentrations were freshly obtained by dilution prior to each experiment. The key physicochemical properties of MB and MO are summarized in Table S1. The pH of the dye solutions was adjusted using standardized 0.1 M hydrochloric acid (HCl) or 0.1 M sodium hydroxide (NaOH) solutions. In addition to synthetic dye solutions, a textile dyeing wastewater sample, containing mixed organic colorants and dissolved inorganic constituents, was collected and treated to assess the practical applicability of the developed sorbent under realistic conditions. The main physicochemical characteristics of the wastewater sample are summarized in Table S2. Pure nitric, hydrochloric, and sulfuric acids (analytical grade) were employed during desorption and regeneration experiments to investigate the reusability and stability of the sorbent.

### 2.2 Synthesis of sorbent

The preparation of the introduced adsorbent in this research work was accomplished through two consecutive steps. In the first one, rice-husk-derived biochar was prepared and chemically activated, while the second step included nitrogen doping and tungstate functionalization to obtain a modified biochar-based composite sorbent.^23,26,34^ Briefly, raw rice husk was thoroughly rinsed with deionized water to remove adhering dust and water-soluble impurities, oven-dried at 105 °C for 24 h, then ground and sieved to obtain a uniform particle size fraction. The dried precursor was subjected to pyrolysis at 600 °C for 2 h under a nitrogen atmosphere (heating rate 10 °C min^−1^) to obtain biochar. After cooling to ambient temperature under nitrogen flow, the produced biochar was ground and stored for the activation step.^23,26^ The pyrolyzed material was then chemically activated using potassium carbonate (K_2_CO_3_) at a weight ratio of 1 : 2 (biochar : K_2_CO_3_). Specifically, biochar (*e.g.*, 10.0 g) was impregnated in an aqueous K_2_CO_3_ solution (K_2_CO_3_: 20.0 g in deionized water, sufficient to fully wet the solid) under continuous stirring for 12 h, followed by drying at 105 °C overnight. The dried impregnated solid was thermally treated at 700 °C for 1 h under nitrogen to promote pore development and activation of the carbon framework. The activated biochar was thoroughly washed with deionized water until the filtrate reached neutral pH, then dried at 100 °C for 12 h and denoted as activated biochar.^23,26^

**Scheme 1:**
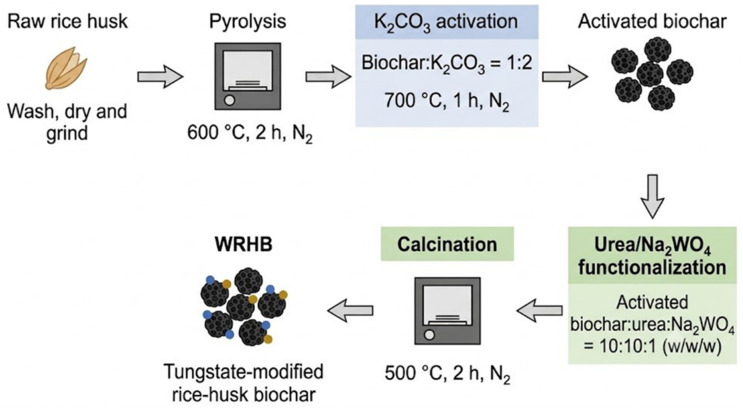
Simplified synthesis pathway of tungstate-modified rice-husk biochar (WRHB). Fully generated with Google Nano Banana and scientifically reviewed and verified by the authors.

In the second step, urea and sodium tungstate were used as precursors for nitrogen doping and tungstate functionalization, respectively.^26,35^ Activated biochar (*e.g.*, 8.0 g) was dispersed in deionized water under stirring, followed by the addition of urea at a mass ratio of 1 : 1 (activated biochar : urea) (*e.g.*, 8.0 g) and sodium tungstate at a mass ratio of 10 : 1 (activated biochar : sodium tungstate) (*e.g.*, 0.8 g). The mixture was stirred for 2 h and then sonicated for 30 min using a homogenizer/probe sonicator to ensure homogeneous impregnation and distribution of the precursors within the biochar matrix. The resulting slurry was dried at 80 °C overnight, gently ground, and subsequently calcined at 500 °C for 2 h under nitrogen to promote stabilization of nitrogen-containing surface functionalities and anchoring of tungstate-derived species onto the carbon surface. The final tungstate-modified rice husk biochar composite (denoted as WRHB) was washed with deionized water to remove weakly bound residues, dried at 60 °C for 24 h, and stored for further characterization and adsorption experiments.^26,35^ The overall synthesis pathway of the WRHB sorbent is schematically illustrated in Scheme 1.

### 2.3 Sorbent characterization

A comprehensive set of analytical techniques was applied to characterize the morphological, structural, and surface properties of the synthesized composite sorbent. The specifications of the employed instruments and the corresponding measurement conditions are described in Section S1 of the SI.

### 2.4 Adsorption experiments

Batch adsorption experiments were conducted to evaluate the adsorption performance of the synthesized tungstate-modified rice husk biochar (WRHB) toward methylene blue (MB) and methyl orange (MO) dyes from aqueous solutions. All adsorption tests were performed in polypropylene centrifuge tubes using a thermostated shaking water bath at controlled temperature and agitation speed. In a typical experiment, a known mass of sorbent (*m*, g) was added to a fixed volume (*V*, L) of dye solution with a predetermined initial concentration (*C*_o_, mg L^−1^). The suspension was agitated at room temperature (25 ± 1 °C) for a specified contact time, after which the solid and liquid phases were separated by filtration prior to analysis. The influence of key operational parameters on the adsorption behavior was systematically investigated. The effect of solution pH was examined over a wide pH range by adjusting the initial pH of the dye solutions using standardized 0.1 M HCl or 0.1 M NaOH solutions. The impact of sorbent dosage was evaluated by varying the amount of sorbent added to the dye solution while keeping other experimental variables constant. Contact time experiments were performed to assess the adsorption kinetics and determine the equilibrium time, whereas the effect of initial dye concentration was studied to evaluate the adsorption isotherm behavior and maximum uptake capacity.

Adsorption kinetic studies were carried out by sampling the dye solutions at predetermined time intervals over a contact time range sufficient to reach equilibrium. The experimental kinetic data were analyzed using commonly applied kinetic models, including pseudo-first-order, pseudo-second-order, and intraparticle diffusion models, as described in the literature.^39–41^ Equilibrium adsorption isotherms were obtained by varying the initial dye concentration at constant temperature, and the resulting data were fitted using Langmuir, Freundlich, and other relevant isotherm models to elucidate the adsorption mechanism and capacity.^41–43^ The adequacy and simplicity of the applied models were assessed using key statistical criteria, including the coefficient of determination (*R*^2^) and the chi-square error function (*χ*^2^), which facilitated a quantitative evaluation of their predictive performance. The corresponding mathematical expressions of the kinetic and isotherm models considered in this work are compiled in Table S3 (ref. 39–43). Thermodynamic parameters of the adsorption process were evaluated by conducting adsorption experiments at different temperatures, and the standard Gibbs free energy, enthalpy, and entropy changes were calculated using established thermodynamic relationships.^44,45^

Desorption and regeneration experiments were conducted to investigate the reusability and stability of the sorbent. After adsorption equilibrium was achieved, the dye-loaded sorbent was separated and treated with appropriate eluents (*e.g.*, acidic solutions) under agitation for a fixed period. The concentration of desorbed dye in the eluate was determined, and the desorption efficiency was calculated accordingly. In addition to experiments conducted with synthetic dye solutions, adsorption tests were also performed using a real textile dyeing wastewater sample to evaluate the practical applicability of the developed sorbent under realistic conditions. The wastewater treatment experiments were carried out following a procedure analogous to that used for synthetic solutions, and the results were compared to assess matrix effects and sorbent robustness.

The concentrations of MB and MO before and after adsorption were determined using a Cary 8454 UV-visible spectrophotometer (Agilent Technologies) by measuring absorbance at *λ*_max_ = 464 nm for MO and *λ*_max_ = 668 nm for MB.^2,3^ Prior to analysis, the samples were filtered through 0.22 µm membrane filters to remove suspended sorbent particles. All adsorption experiments were conducted in triplicate, and the mean values were reported; only results with a relative error of ≤5% were considered acceptable. The adsorption capacity (*q*_e_, mg g^−1^), removal efficiency (%), and other relevant parameters were calculated using standard mass-balance equations, which are provided in the SI.

## 3 Results & discussion

### 3.1 Sorbent characteristics

#### 3.1.1 XRD patterns

**Fig. 1:**
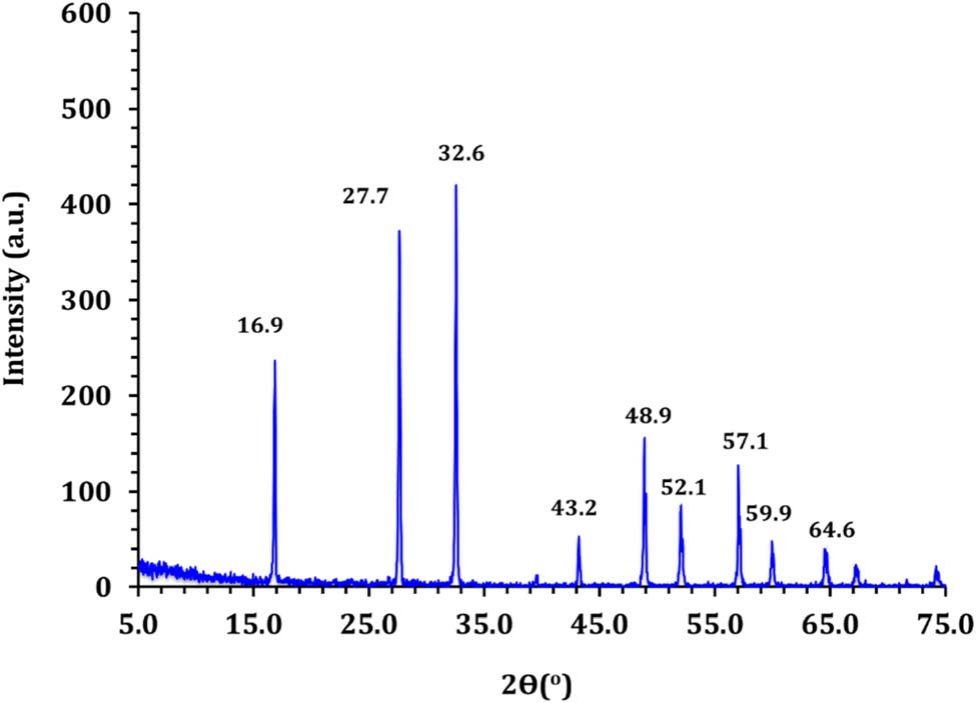
XRD pattern of the tungstate-modified rice husk biochar composite.

The crystalline structure and phase composition of the synthesized tungstate-modified rice husk biochar composite were investigated by X-ray diffraction (XRD), and the resulting diffraction pattern is presented in Fig. 1. The pattern exhibits a low-intensity, broad amorphous background spanning the 2*θ* range of approximately 15–30°, which is characteristic of carbonaceous materials derived from lignocellulosic biomass and reflects the disordered turbostratic structure of the rice-husk-derived biochar framework.^17,21^ The presence of this amorphous contribution confirms that the carbon matrix remains largely non-crystalline after chemical activation, nitrogen doping, and tungstate functionalization, thereby serving as a stable supporting scaffold for the incorporated inorganic species. Superimposed on the amorphous carbon background, distinct diffraction reflections are observed at 2*θ* = 16.9°, 27.7°, 32.6°, 43.2°, 48.9°, 52.0°, 57.1°, 59.9°, and 64.5°. These reflections are assigned to crystalline Na_2_WO_4_ according to PDF No. 00-012-0772, confirming the formation of tungstate-derived crystalline domains within the biochar matrix. The coexistence of distinct tungstate crystalline reflections with the amorphous carbon baseline highlights the hybrid nature of the composite, consisting of a disordered biochar matrix decorated with well-dispersed inorganic tungstate domains. Importantly, no additional diffraction peaks associated with common mineral impurities are detected, suggesting that sodium tungstate oxide is the dominant crystalline phase present in the material, in agreement with previously reported tungstate–biochar composite systems.^33–35^ From a functional perspective, the retained amorphous carbon framework provides abundant defect sites and π-electron-rich domains, while the crystalline sodium tungstate oxide phase introduces polar and coordinatively active surface sites capable of participating in electrostatic interactions and surface complexation with dye molecules. This synergistic structural configuration establishes a robust physicochemical basis for the enhanced adsorption performance toward methylene blue and methyl orange discussed in subsequent sections.^33–35^

#### 3.1.2 FTIR spectra

**Fig. 2:**
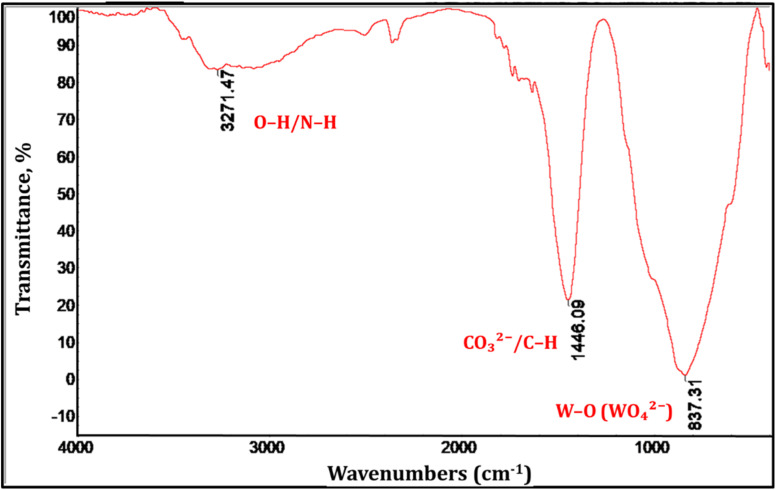
FTIR spectrum of the tungstate-modified rice husk biochar composite.

The surface functional groups and chemical characteristics of the WRHB sorbent were investigated using Fourier transform infrared (FTIR) spectroscopy, and the resulting spectrum is presented in Fig. 2. The broad absorption band centered at 3271.47 cm^−1^ is assigned predominantly to O–H stretching vibrations of surface hydroxyl groups and adsorbed water, with a possible overlapping contribution from N–H functionalities introduced during urea treatment. These polar functionalities can participate in hydrogen-bonding and other specific interactions with dye molecules.^24–26^ The prominent band at 1446.09 cm^−1^ is attributed mainly to carbonate-related vibrations, with a possible overlapping contribution from C–H deformation within the carbon framework. The presence of carbonate-related species is consistent with the use of K_2_CO_3_ during chemical activation. In the lower-wavenumber region, the intense band at 837.31 cm^−1^ is assigned to W–O stretching vibrations within WO_4_^2−^ units.^24–26^ This band supports the incorporation of tungstate-derived domains into the carbon matrix, in agreement with the crystalline tungstate phase identified by XRD (Fig. 1). Collectively, the hydroxyl- and nitrogen-containing functionalities, carbonate-related groups, and tungstate-derived W–O domains establish a chemically heterogeneous surface containing potential interaction sites for MB and MO adsorption.

#### 3.1.3 SEM-EDS analyses

**Fig. 3:**
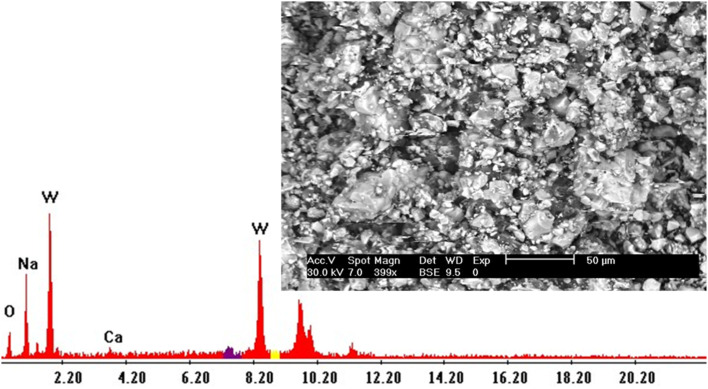
SEM-EDX spectrum of the tungstate-modified rice husk biochar composite.

The surface morphology and compositional heterogeneity of the WRHB sorbent were examined by scanning electron microscopy coupled with energy-dispersive X-ray spectroscopy (SEM-EDS), as shown in Fig. 3. The SEM micrographs reveal an irregular and heterogeneous texture composed of agglomerated particles with rough, fractured surfaces and abundant micro-cavities, which is consistent with the typical morphological evolution of chemically activated biochars derived from lignocellulosic precursors and is beneficial for enhancing surface accessibility and diffusion of adsorbate molecules toward internal sites.^23,38^ In addition, the backscattered electron (BSE) images exhibit clear brightness contrast across the particle surfaces, evidencing compositional heterogeneity and supporting the presence of a carbonaceous matrix decorated with domains of higher average atomic number, rather than a chemically uniform carbon surface.^26,34,35^ The accompanying EDS spectrum (Fig. 3) confirms the presence of tungsten (W) together with oxygen (O) and sodium (Na), providing direct compositional evidence for the successful incorporation of tungstate-derived species originating from the sodium tungstate precursor during the functionalization step.^34,35^ Minor calcium (Ca) signals are also detected, which can reasonably be attributed to the inherent mineral fraction commonly associated with rice-husk-derived carbonaceous materials. It should be emphasized that EDS is applied here primarily for qualitative verification of the incorporated inorganic species and their presence on the examined surface regions, rather than for rigorous bulk quantification. Overall, the combined SEM-EDS results corroborate the formation of a heterogeneous biochar-based composite decorated with tungstate-related domains, in good agreement with the crystalline tungstate features identified by XRD (Fig. 1).

#### 3.1.4 Textural properties

**Fig. 4:**
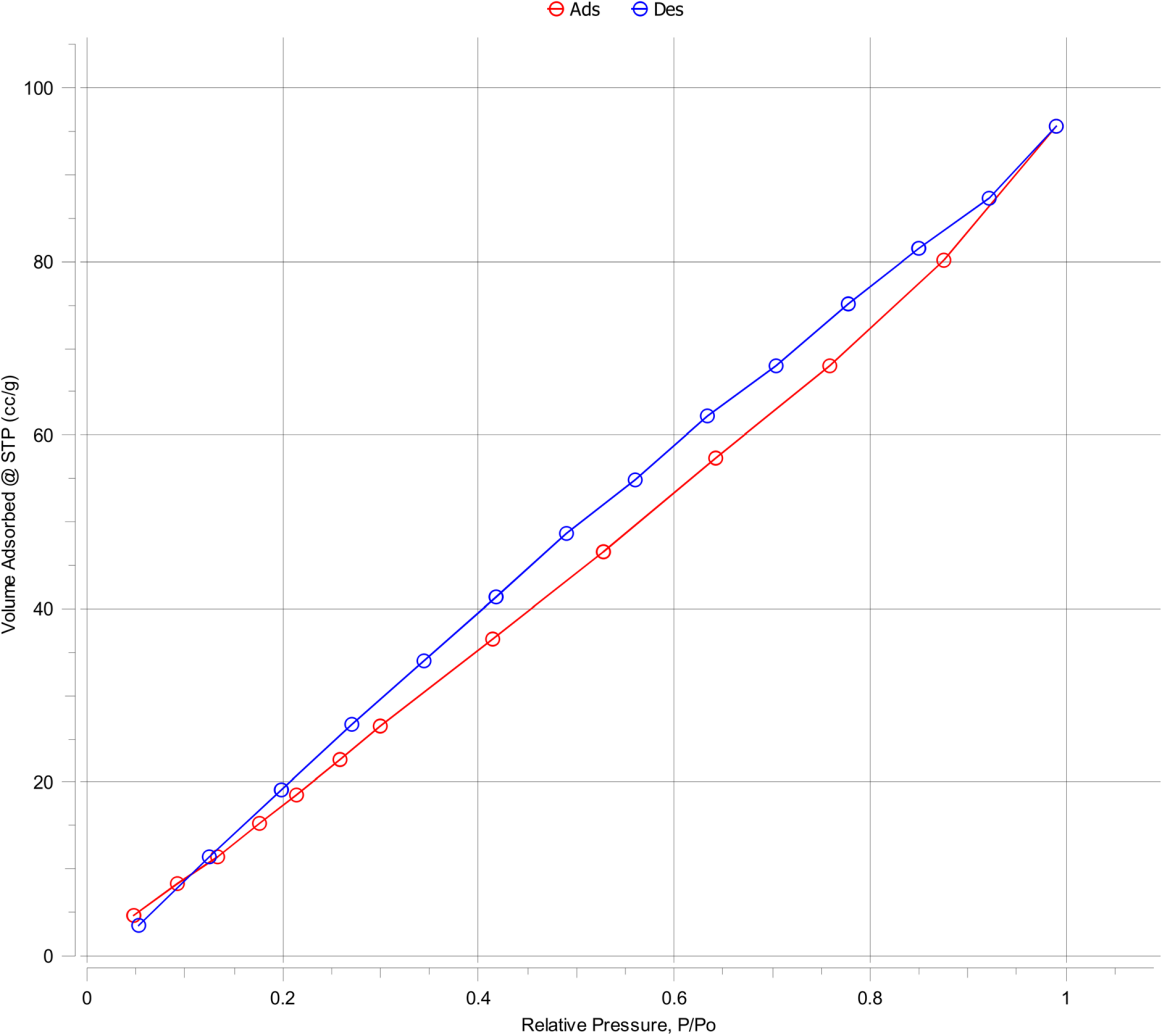
Nitrogen adsorption–desorption isotherm of WRHB.

The textural properties of the WRHB composite were evaluated by nitrogen adsorption–desorption measurements at 77 K, and the corresponding isotherm is presented in Fig. 4. The observed adsorption behavior reflects the development of an accessible porous architecture induced by potassium carbonate activation. During thermal treatment, K_2_CO_3_ promotes controlled carbon gasification and partial etching of the carbon framework, generating new pores and enlarging existing ones within the biochar matrix.^23,26^ This activation mechanism contributes to the formation of a structurally stable yet porous network suitable for adsorption applications. The WRHB sorbent exhibits a Brunauer–Emmett–Teller (BET) specific surface area of 145.4 m^2^ g^−1^ (Table 1), indicating that the applied activation strategy effectively enhanced surface accessibility while preserving the structural integrity of the carbon backbone. In addition to surface area enhancement, the composite displays a total pore volume of 0.148 cm^3^ g^−1^ (Table 1), confirming the presence of a well-developed pore system capable of accommodating organic dye molecules. For adsorption processes involving relatively large molecular species such as methylene blue and methyl orange, pore accessibility and effective diffusion pathways often play a more decisive role than extremely high surface areas alone.^20,26^ In this context, the moderate surface area combined with an appreciable pore volume provides a favorable balance between adsorption-site availability and mass-transfer efficiency. Such a structural configuration is advantageous for facilitating rapid dye diffusion toward active sites while minimizing internal transport limitations, thereby contributing positively to the overall adsorption performance of the WRHB composite.^20,26,38^

**Table 1:** Surface properties of the WRHB sorbent

*S*_BET_ (m^2^ g^−1^)	*V*_P_ (cm^3^ g^−1^)	*D*_H_ (nm)
145.4	0.148	3.80

#### 3.1.5 Particle size analysis

**Fig. 5:**
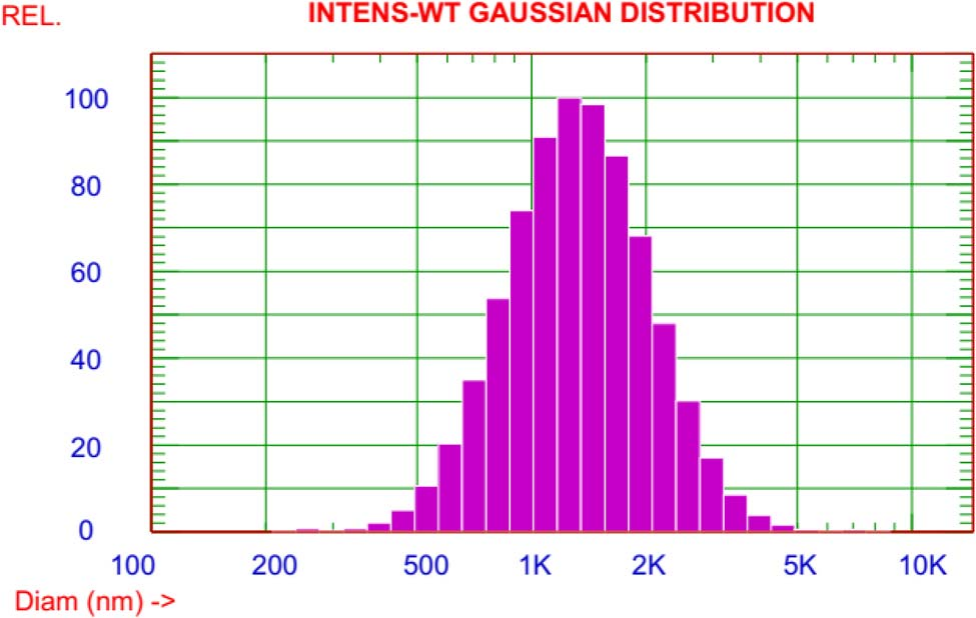
Particle size distribution of the WRHB sorbent.

The hydrodynamic particle size distribution of the WRHB sorbent was determined using dynamic light scattering (DLS), and the corresponding intensity-weighted distribution is presented in Fig. 5. The results reveal a broad particle size distribution with a mean hydrodynamic diameter of approximately 1451 nm, indicating that the sorbent particles predominantly exist as micron-scale agglomerates when dispersed in aqueous media. Such aggregation behavior is frequently observed in biochar-based and carbonaceous composite sorbents due to strong interparticle interactions, surface heterogeneity, and the inherently irregular morphology of pyrolyzed carbon materials.^1,2^ The measured polydispersity index (PDI) of 0.185 reflects a moderately heterogeneous size distribution, which is consistent with the rough surface texture and compositional heterogeneity identified in the SEM analysis. Cumulative distribution analysis further shows that 50% and 90% of the dispersed particles exhibit hydrodynamic diameters below approximately 1322 nm and 2294 nm, respectively, confirming the presence of a distribution dominated by aggregated domains rather than discrete nanoscale primary particles. It should be noted that DLS determines the hydrodynamic diameter of particles in suspension, which includes solvation layers and agglomerated clusters formed under dispersion conditions, rather than the true primary particle size.^26,27^ Therefore, the DLS results do not contradict the morphological observations obtained from SEM or the porosity data derived from N_2_ adsorption measurements. In the context of batch adsorption experiments, micron-scale aggregation is not inherently detrimental, as adsorption performance is governed primarily by accessible surface chemistry and internal pore structure rather than colloidal stability alone.^26,35^

#### 3.1.6 Point of zero charge (pH_pzc_) analysis

**Fig. 6:**
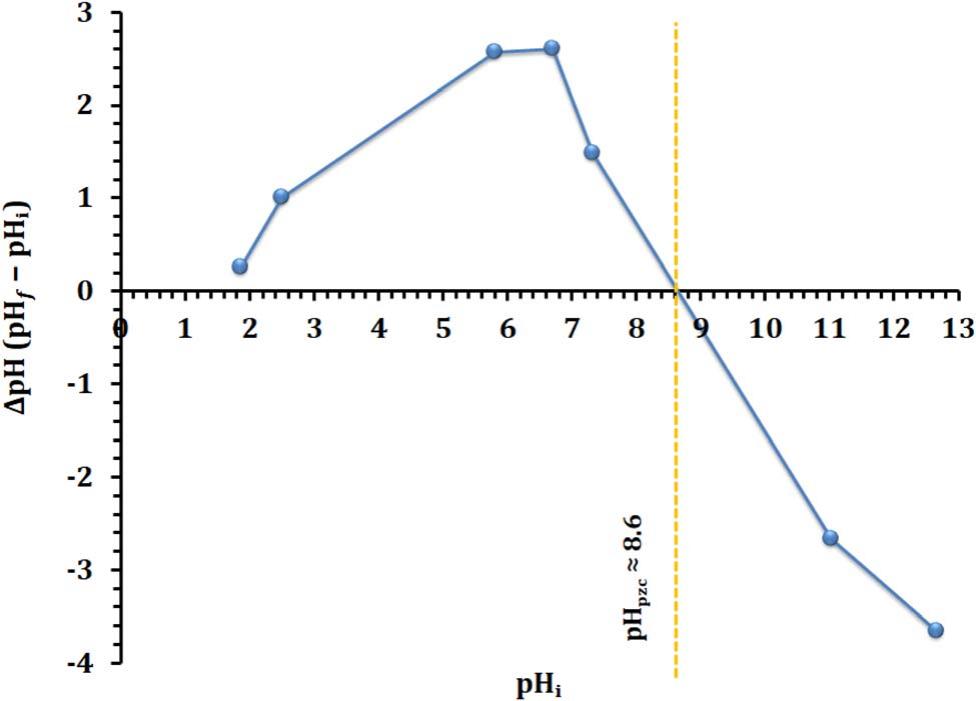
Determination of the point of zero charge (pH_pzc_) of WRHB using the pH-drift method.

The point of zero charge (pH_pzc_) of the WRHB sorbent was determined using the pH-drift method, and the corresponding ΔpH (pH_f_ − pH_i_) profile is presented in Fig. 6. The curve intersects the zero line at approximately pH_pzc_ = 8.6, indicating that the net surface charge of the composite becomes electrically neutral at this pH value. Below pH 8.6, the surface is predominantly positively charged due to protonation of oxygen-containing and heteroatom surface functionalities, whereas above this value progressive deprotonation results in a negatively charged surface.^46,47^ The relatively high pH_pzc_ observed for WRHB reflects the chemically modified nature of the composite. Alkali activation introduces basic surface sites and residual alkaline species that can shift the surface-charge neutrality point toward the alkaline region.^46,47^ In addition, the incorporation of tungstate-derived inorganic domains contributes metal–oxygen surface groups capable of influencing charge distribution and surface protonation equilibria.^46–48^ The combined effects of carbon activation and inorganic functionalization therefore account for the elevated pH_pzc_ compared with unmodified biochars. From an adsorption standpoint, this charge-switching behavior provides a mechanistic framework for interpreting dye uptake. Under conditions where the solution pH is below the pH_pzc_, the positively charged surface enhances electrostatic attraction toward anionic methyl orange molecules, while adsorption of cationic methylene blue becomes less electrostatically favorable and instead proceeds predominantly through non-electrostatic interactions such as π–π stacking, hydrogen bonding, pore filling, and possible surface complexation.^46–48^ Consequently, the experimentally determined pH_pzc_ offers a fundamental basis for understanding the pH-dependent adsorption trends discussed in the subsequent sections.

Collectively, the XRD, SEM-EDS, FTIR, textural, particle size, and pH_pzc_ analyses provide a comprehensive and internally consistent picture of the synthesized WRHB sorbent as a chemically heterogeneous, structurally stable biochar-based composite. The material combines an amorphous carbon framework with finely dispersed tungstate domains, accessible porosity, polar surface functionalities, and tunable surface-charge characteristics. This synergistic combination of structural, chemical, and interfacial properties establishes a robust physicochemical foundation for efficient adsorption of both anionic and cationic dyes and underpins the adsorption behavior and mechanistic interpretations presented in the following sections.

### 3.2 Batch investigation

**Fig. 7:**
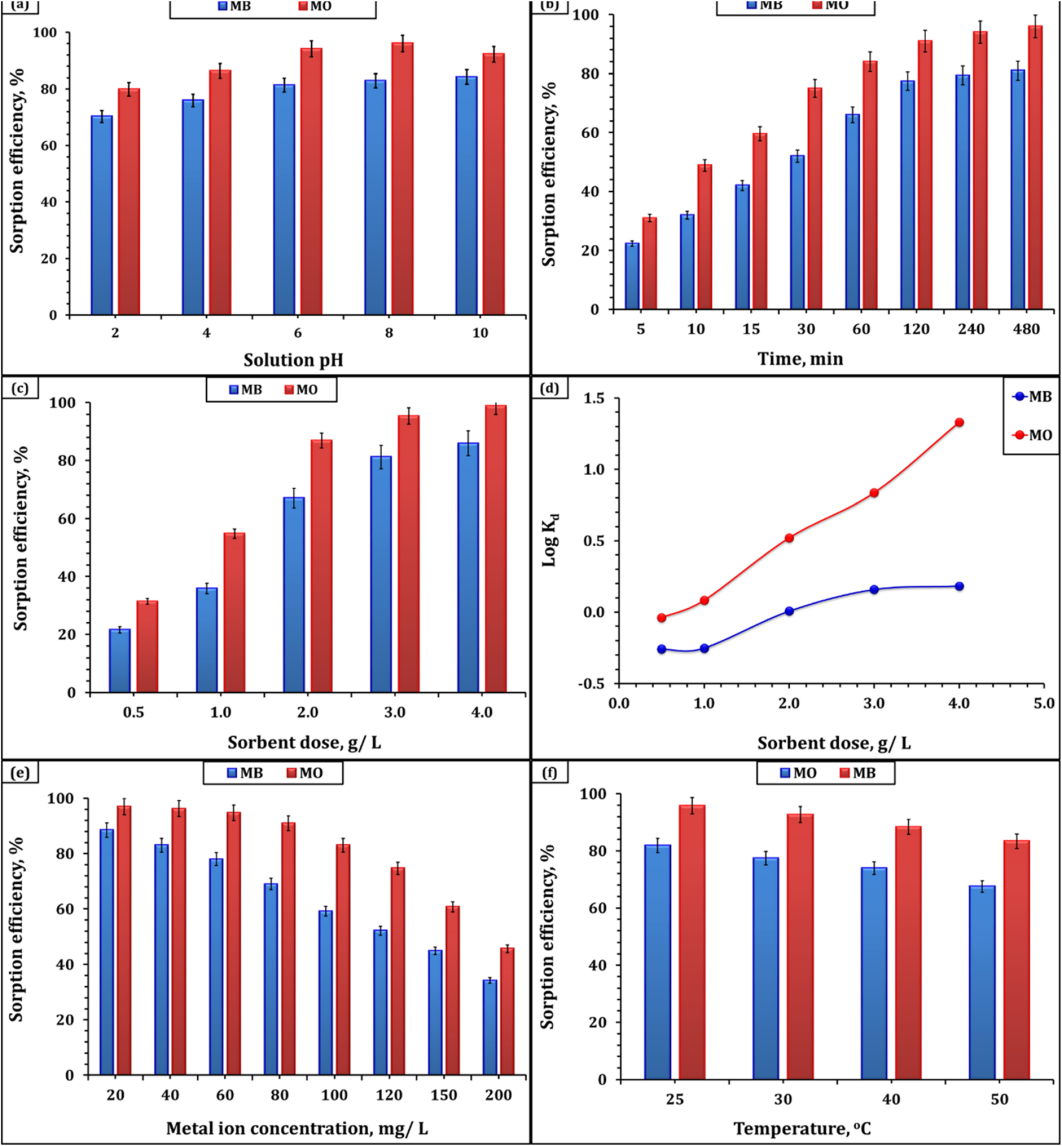
Sorption tendency of MB, and MO depending on (a) solution pH (WRHB dosage: 3.0 g L^−1^; initial concentration: 50 mg L^−1^; room temperature; and time: 240 min), (b) shaking time (room temperature; WRHB dosage of 3.0 g L^−1^; initial concentration of 50 mg L^−1^; pH 6.02), (c) WRHB dosage (pH 6.02; time of 240 min; initial concentration: 50 mg L^−1^; and room temperature), (d) distribution coefficient (log *K*_d_) (pH 6.02; time of 240 min; initial concentration: 50 mg L^−1^; and room temperature), (e) initial concentration (time: 240 min; room temperature; pH 6.01; and WRHB dosage: 3.0 g L^−1^), and (f) temperature (pH 6.02; time of 240 min; initial concentration: 50 mg L^−1^; WRHB dosage: 3.0 g L^−1^).

The adsorption performance of the synthesized composite sorbent towards MB and MO was examined systematically under a variety of operating conditions, encompassing solution pH (2–10), contact time (5–480 minutes), sorbent dosage (0.5–4 g L^−1^), initial dye concentration (20–200 mg L^−1^), and temperature (25–50 ± 1 °C), as depicted in Fig. 7. This thorough assessment aimed to clarify the real-world adsorption properties of the sorbent under various conditions and to determine the best operating settings that achieve maximum dye removal effectiveness.

Solution pH plays a decisive role in governing the adsorption efficiency of both methylene blue (MB) and methyl orange (MO), as illustrated in Fig. 7a. Within the investigated range (pH 2–10), MO removal efficiency increased sharply from 79.9% at pH 2 (*q*_e_ = 13.3 mg g^−1^) to a maximum of 96.1% at pH 8 (*q*_e_ = 16.0 mg g^−1^), followed by a slight decline to 92.3% at pH 10 (*q*_e_ = 15.4 mg g^−1^). This behavior is consistent with the anionic nature of MO and the sorbent's point of zero charge (pH_pzc_ ≈ 8.6). Under acidic conditions below the pH_pzc_, the positively charged surface promotes strong electrostatic attraction toward MO anions, whereas progressive surface deprotonation at alkaline pH reduces this electrostatic driving force, accounting for the modest decrease in removal efficiency beyond neutrality.^2,27^ In contrast, MB adsorption exhibited a gradual and nearly monotonic increase over the same pH range, rising from 70.3% at pH 2 (*q*_e_ = 11.7 mg g^−1^) to 84.3% at pH 10 (*q*_e_ = 14.1 mg g^−1^). The sustained uptake of MB even under acidic conditions indicates that non-electrostatic interactions contribute significantly to its adsorption. These interactions include π–π stacking between the aromatic rings of MB and the conjugated carbon framework of the sorbent, hydrogen bonding with oxygen- and nitrogen-containing surface functionalities, and pore filling within the mesoporous network.^15,26^ Because these mechanisms are less sensitive to variations in surface charge, MB adsorption displays a smoother and more gradual pH dependence compared with MO. Taken together, the contrasting adsorption behaviors of MB and MO under varying pH conditions highlight the multifunctional binding capability of the WRHB composite. While MO adsorption is predominantly governed by electrostatic attraction modulated by surface charge, MB uptake is strongly influenced by structural and chemical interactions that remain operative across the entire pH range.^26,28,38,49^ This complementary adsorption behavior is fully consistent with the physicochemical characterizations presented earlier, which revealed a porous aromatic framework combined with a pH-dependent surface charge distribution.

Contact time is a critical factor influencing the adsorption efficiency of both methylene blue (MB) and methyl orange (MO), as presented in Fig. 7b. The adsorption profiles exhibit a rapid initial uptake followed by a slower approach to equilibrium, which is characteristic of adsorption on porous, heterogeneous carbon-based sorbents. For MB, removal efficiency increased from 22.3% at 5 min (*q*_*t*_ = 3.7 mg g^−1^) to 66.0% at 60 min (*q*_*t*_ = 11.0 mg g^−1^), and then gradually reached 81.0% at 480 min (*q*_*t*_ = 13.5 mg g^−1^). A more pronounced uptake was observed for MO, which increased from 31.0% at 5 min (*q*_*t*_ = 5.2 mg g^−1^) to 84.0% at 60 min (*q*_*t*_ = 14.0 mg g^−1^), before approaching 96.0% at 480 min (*q*_*t*_ = 16.0 mg g^−1^). The steep increase during the first hour indicates that readily accessible external sites and pore-mouth regions are rapidly occupied due to strong dye-surface affinity, whereas the subsequent slower stage is attributed to gradual diffusion into less accessible pore domains and progressive saturation of the remaining active sites.^34,38,49^ From an application standpoint, a contact time of 120 min is sufficient to achieve near-equilibrium uptake, while 240 min provides conservative equilibrium conditions for both dyes and minimizes uncertainty in subsequent isotherm and thermodynamic analyses. The different uptake rates of MB and MO can be rationalized by their molecular characteristics and the chemically heterogeneous surface of WRHB. Under the working pH conditions, MO (an anionic dye) benefits from stronger electrostatic attraction to positively charged sites, promoting faster initial site occupation, whereas MB adsorption relies more heavily on non-electrostatic interactions such as π–π stacking with the aromatic carbon framework and hydrogen bonding with oxygen-/nitrogen-containing surface groups, which can exhibit a comparatively slower approach to equilibrium. Overall, the observed two-stage uptake behavior is consistent with the sorbent's textural and chemical characteristics, where accessible porosity supports rapid surface binding followed by slower intraparticle diffusion into internal adsorption domains.^26,34,38,49^

Sorbent dosage exerts a pronounced influence on adsorption efficiency and equilibrium capacity, as illustrated in Fig. 7c. Increasing the WRHB dose from 0.5 to 4.0 g L^−1^ significantly enhanced dye removal for both methylene blue (MB) and methyl orange (MO). For MB, removal efficiency increased from 21.6% to 85.9%, whereas MO removal rose from 31.4% to 98.8% over the same dosage range. This improvement reflects the progressive increase in available adsorption sites and accessible surface area as additional sorbent mass is introduced into the system. In contrast, the adsorption capacity (*q*_e_) decreased with increasing dose, declining from 21.6 to 10.7 mg g^−1^ for MB and from 31.4 to 12.4 mg g^−1^ for MO. This inverse relationship between removal efficiency and adsorption capacity is commonly observed in batch adsorption systems and arises from the balance between available binding sites and the fixed concentration of dye molecules in solution. At lower sorbent dosages, active sites are comparatively limited, leading to higher site utilization and greater adsorption capacity per unit mass. As the dosage increases, the number of adsorption sites exceeds the number of dye molecules present, resulting in partial site underutilization and a reduction in qe, even though overall removal efficiency continues to improve.^2,3,14,38^ The distribution coefficient (*K*_d_) exhibited a positive correlation with sorbent dosage, particularly for MO, indicating stronger affinity of the composite toward anionic dye species (Fig. 7d). This behavior is consistent with the sorbent's surface-charge characteristics and heterogeneous functional group distribution, which favor electrostatic and specific interactions with negatively charged species under the working conditions. From an operational perspective, a dosage of 3.0 g L^−1^ represents a balanced condition, achieving high removal efficiency while maintaining reasonable adsorption capacity. Excessively high dosages may lead to material underutilization and increased operational cost, whereas insufficient dosage compromises overall removal performance. The observed trends are in agreement with previous investigations on carbonaceous and composite sorbents, where dosage optimization has been identified as a key parameter for balancing adsorption efficiency, cost-effectiveness, and process sustainability in batch systems.^24,26,38,49^

The initial dye concentration significantly influences both the thermodynamic driving force for adsorption and the extent of surface-site saturation, as illustrated in Fig. 7e. Increasing the concentration from 20 to 200 mg L^−1^ resulted in a gradual decrease in removal efficiency for both methylene blue (MB) and methyl orange (MO), while the equilibrium adsorption capacity (*q*_e_) increased correspondingly. For MB, removal efficiency declined from 88.5% (*q*_e_ = 5.9 mg g^−1^) at 20 mg L^−1^ to 34.3% (*q*_e_ = 22.8 mg g^−1^) at 200 mg L^−1^. A similar trend was observed for MO, where efficiency decreased from 97.0% (*q*_e_ = 6.5 mg g^−1^) to 45.7% (*q*_e_ = 30.5 mg g^−1^) across the same concentration range. This inverse relationship between removal efficiency and adsorption capacity reflects the interplay between available binding sites and solute concentration.^2,3,26,38^ At lower concentrations, the number of active sites greatly exceeds the number of dye molecules, resulting in high removal efficiency but relatively modest adsorption capacity per unit mass. As the concentration increases, the enhanced concentration gradient provides a stronger mass-transfer driving force, promoting greater adsorption per gram of sorbent and increasing *q*_e_. However, because the number of accessible sites remains finite, progressive occupation leads to partial saturation, thereby reducing overall removal efficiency. The tendency toward plateauing at higher concentrations further indicates the gradual saturation of available adsorption sites, consistent with a finite-capacity adsorption system.^17,26,38^ From a practical perspective, an initial concentration range of 40–60 mg L^−1^ offers a balanced operating window, ensuring high removal efficiency while maintaining appreciable adsorption capacity. Such optimization is particularly relevant for routine batch studies and treatment of real wastewater streams, where dye concentrations can vary substantially. The observed behavior aligns with established adsorption theory for carbonaceous and composite sorbents, in which mesoporous structures accommodate increasing dye loads but remain constrained by limited active-site availability.^17,26,38^

Temperature exerts a significant influence on adsorption efficiency, as illustrated in Fig. 7f. Both methylene blue (MB) and methyl orange (MO) exhibited a gradual decrease in uptake as temperature increased from 25 to 50 °C. For MB, removal efficiency declined from 81.9% (*q*_e_ = 13.7 mg g^−1^) at 25 °C to 67.5% (*q*_e_ = 11.3 mg g^−1^) at 50 °C. A comparable trend was observed for MO, where removal decreased from 95.8% (*q*_e_ = 16.0 mg g^−1^) to 83.4% (*q*_e_ = 13.9 mg g^−1^) over the same temperature range. The reduction in adsorption performance at elevated temperatures suggests that the adsorption process is thermodynamically unfavorable at higher thermal energy levels. Increased molecular mobility at higher temperatures can weaken dye–sorbent interactions and reduce the residence time of dye molecules at active sites, thereby diminishing overall uptake. Conversely, lower temperatures favor stronger binding interactions and enhanced adsorption efficiency, indicating that the process is likely exothermic in nature.^38,50,51^ These observations are consistent with the structural and chemical characteristics of the WRHB composite, which features a mesoporous carbon framework and diverse surface functional groups capable of effective dye binding under ambient conditions. From a practical perspective, achieving high removal efficiencies at room temperature is advantageous, as it eliminates the need for external heating and reduces operational costs. Such energy-efficient performance enhances the applicability of the sorbent in real wastewater treatment systems, where process simplicity and sustainability are essential considerations.^38,50,51^

In summary, the batch adsorption investigation demonstrates that the performance of the synthesized WRHB composite toward methylene blue (MB) and methyl orange (MO) is governed by the interplay between solution chemistry, sorbent dosage, concentration gradients, and operating temperature. Optimal removal efficiencies were achieved under near-neutral pH conditions, contact times of 120–240 minutes, a sorbent dosage of 3.0 g L^−1^, initial dye concentrations of 40–60 mg L^−1^, and ambient temperature. These parameters collectively balance electrostatic attraction, surface-site availability, and non-electrostatic interactions such as π–π stacking and hydrogen bonding, ensuring both high removal efficiency and meaningful adsorption capacity. The observed trends—namely strong pH dependence, rapid initial uptake followed by diffusion-controlled equilibration, inverse relationships between dosage and adsorption capacity, concentration-driven site saturation, and reduced adsorption at elevated temperatures—are characteristic of porous carbonaceous and composite adsorption systems. Comparable behaviors have been reported for activated and heteroatom-modified rice husk biochars as well as inorganic–biochar hybrid materials designed for dual dye removal.^17,26,34,38^ These parallels indicate that the WRHB composite follows established adsorption principles while simultaneously offering multifunctional binding capacity for both cationic and anionic dyes within a single material platform. Collectively, the variable-parameter analysis establishes a coherent mechanistic foundation for subsequent kinetic, equilibrium, and thermodynamic modeling. The ability to achieve high removal efficiencies at room temperature, combined with adaptability toward mixed dye systems, positions the present composite alongside other proven carbon-based and hybrid sorbents that have demonstrated practical relevance in wastewater treatment applications.

### 3.3 Adsorption kinetics, equilibrium, and thermodynamic evaluation

Building upon the parameter-dependent adsorption trends discussed above, a comprehensive kinetic, equilibrium, and thermodynamic analysis was undertaken to quantitatively characterize the adsorption behavior of methylene blue and methyl orange on the WRHB composite. Kinetic modeling was employed to clarify the rate-controlling mechanisms and time-dependent uptake characteristics, while equilibrium isotherm analysis was conducted to describe adsorption capacity, surface affinity, and site heterogeneity under varying concentration conditions. In parallel, thermodynamic parameters were evaluated to determine the feasibility, spontaneity, and energetic nature of the adsorption process. Collectively, these complementary approaches provide a rigorous quantitative framework for interpreting the sorbent's performance in relation to its hybrid carbon–inorganic architecture, functional surface chemistry, mesoporous texture, and alkaline pH_pzc_. This analysis establishes a solid foundation for the mechanistic discussion that follows.

#### 3.3.1 Uptake kinetics

**Fig. 8:**
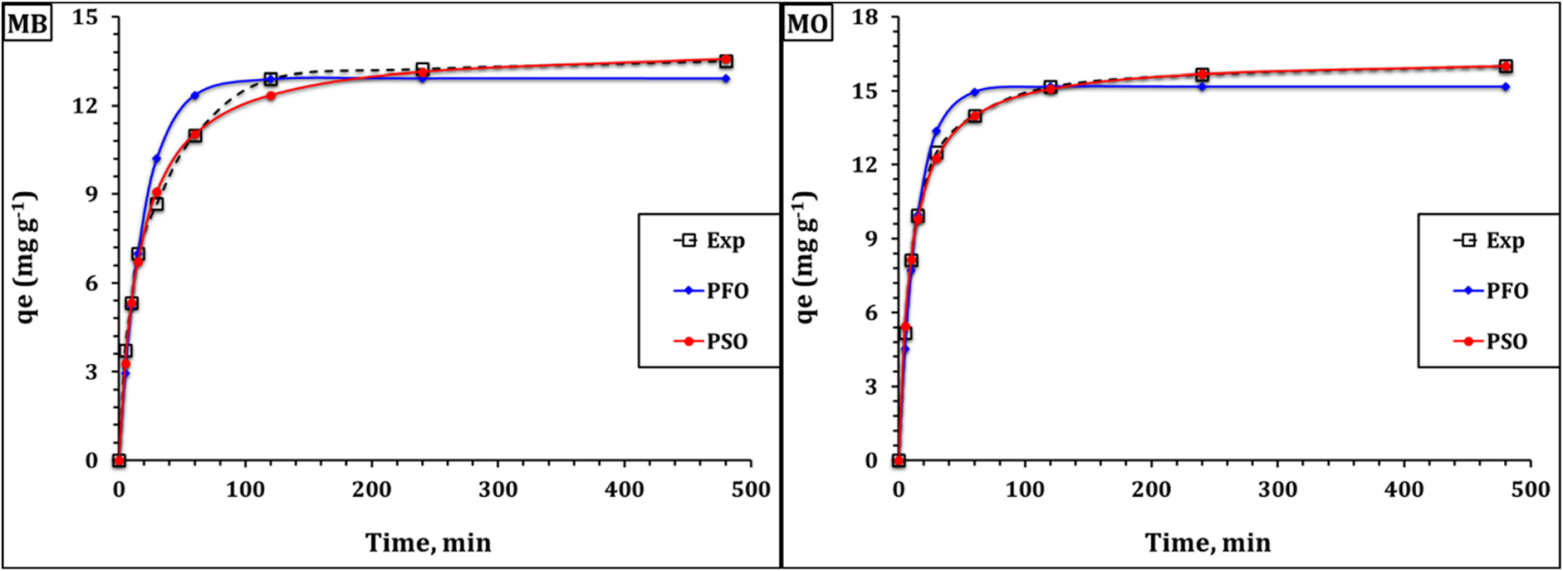
The kinetic curve of MB and MO uptake process (solution pH 6.02; temperature of 25 ± 1 °C; initial concentration of 50 mg L^−1^; WRHB dosage of 3.0 g L^−1^).

The adsorption kinetics of methylene blue (MB) and methyl orange (MO) onto the WRHB sorbent were evaluated using the pseudo-first-order (PFO), pseudo-second-order (PSO), and Weber–Morris intraparticle diffusion (IPD) models. The variation of adsorption capacity (*q*_*t*_) with contact time was monitored over 5–480 min at pH 6.02, room temperature, an initial dye concentration of 50 mg L^−1^, and a sorbent dosage of 3.0 g L^−1^. The kinetic profiles and model fittings are presented in Fig. 8, and the corresponding parameters are summarized in Table 2.

**Table 2:** The values of the applied kinetics model parameters

	MB	MO
**Pseudo first-order model**		
*q*_1_ (mg g^−1^)	12.9	15.2
*k*_1_ (min^−1^)	0.052	0.071
*R* ^2^	0.95	0.97
*X* ^2^	0.61	0.29

**Pseudo second-order model**		
*q* ^2^ (mg g^−1^)	14.0	16.3
*k*_2_ (min^−1^)	0.004	0.006
*h* (mg g^−1^ min^−1^)	0.9	1.6
*t*_1/2_ (min)	16.3	10.0
*R* ^2^	0.99	0.99
*X* ^2^	0.11	0.02

According to the PFO model, the calculated equilibrium adsorption capacities (*q*_1_) were 12.9 mg g^−1^ for MB and 15.2 mg g^−1^ for MO, with rate constants (*k*_1_) of 0.052 min^−1^ and 0.071 min^−1^, respectively. Although acceptable linearity was obtained (*R*^2^ = 0.95 for MB and 0.97 for MO), the relatively elevated chi-square (*χ*^2^) values (0.61 for MB and 0.29 for MO) reveal noticeable deviations between calculated and experimental adsorption profiles. This indicates that the PFO model does not satisfactorily capture the full adsorption behavior across the investigated time domain. In contrast, the PSO model provides a markedly improved representation of the adsorption kinetics for both dyes. The calculated equilibrium capacities (*q*_2_) increase to 14.0 mg g^−1^ for MB and 16.3 mg g^−1^ for MO, closely matching the experimentally observed near-equilibrium values. Furthermore, the PSO model yields higher correlation coefficients (*R*^2^ = 0.99 for both dyes) and significantly lower chi-square values (0.11 for MB and 0.02 for MO), confirming its superior predictive capability. The dominance of the PSO model suggests that adsorption is governed primarily by the availability of active surface sites and the strength of sorbate–sorbent interactions rather than simple concentration-dependent first-order kinetics.^39–41^ Additional insight is obtained from the PSO-derived kinetic parameters. The rate constants (*k*_2_) were 0.004 min^−1^ for MB and 0.006 min^−1^ for MO, while the calculated initial adsorption rates (*h*) were 0.9 mg g^−1^ min^−1^ for MB and 1.6 mg g^−1^ min^−1^ for MO. The half-equilibrium times (*t*_0.5_) of 16.3 min for MB and 10.0 min for MO further confirm the faster uptake of MO under identical conditions. This behavior is consistent with the stronger electrostatic affinity of the WRHB surface toward anionic dye species, as previously discussed in the pH-dependent adsorption analysis.

To examine potential mass-transfer limitations, the Weber–Morris intraparticle diffusion model was applied. The IPD plots (Fig. S1) and parameters (Table S4) reveal intraparticle diffusion constants (*k*_i_) of 0.60 mg g^−1^ min^−1/2^ for MB and 0.54 mg g^−1^ min^−1/2^ for MO, with high correlation coefficients (*R*^2^ = 0.98 for both dyes). However, the non-zero intercept values (*C* = 7.1 for MB and 4.9 for MO) indicate that the plots do not pass through the origin, demonstrating that intraparticle diffusion is not the sole rate-controlling step.^39–41^ The presence of significant intercept values confirms the contribution of boundary-layer (film diffusion) effects, particularly for MB. Such multi-step kinetic behavior—characterized by rapid surface adsorption followed by diffusion into internal pores—is typical for heterogeneous porous carbon-based and hybrid sorbents.^17,26,34,38^

Comparable kinetic behavior has been widely documented for related sorbent systems. Activated biocarbon derived from birchwood pellets^2^ and tamarind seed-based activated carbon and chitosan composites^3^ exhibited adsorption kinetics better described by the PSO model, indicating that surface-site interactions dominate the rate process. Similarly, activated carbons prepared from agricultural residues^14^ demonstrated rapid initial uptake followed by diffusion-controlled stages. Reviews on nitrogen-doped biochars^24^ and experimental studies on N-doped bagasse-derived biochar^26^ further confirm that heteroatom functionalization enhances adsorption rates and promotes PSO-dominated kinetics through strengthened dye–surface interactions. Nitrogen-rich seaweed-derived carbon materials capable of simultaneous cationic and anionic dye removal also showed rapid surface adsorption followed by intraparticle diffusion contributions.^28^ In hybrid inorganic–carbon systems, WO_3_@TiO_2_/biochar heterojunctions,^34^ Mo–Bi_2_WO_6_/WO_3_/biochar composites,^35^ WO_3_-based biomass composites,^36^ and Z-scheme biochar-based heterojunctions^37^ displayed similar PSO-controlled kinetics accompanied by non-zero IPD intercepts, indicating the coexistence of surface adsorption and diffusion limitations. Rice husk-derived magnetic biochar used for dual dye removal likewise followed PSO kinetics with multi-step diffusion behavior.^38^ These parallels demonstrate that the kinetic performance of the WRHB composite is fully consistent with established activated, nitrogen-modified, and tungsten-containing biochar systems.

Overall, the kinetic analysis confirms that MO is adsorbed more rapidly and to a slightly greater extent than MB, and that the adsorption process proceeds through a sequential pathway involving rapid surface binding followed by diffusion-controlled stages. The strong agreement between kinetic modeling results and the trends observed in the parameter-effect studies reinforces the reliability of the proposed adsorption mechanism and establishes a robust quantitative basis for the subsequent equilibrium isotherm and thermodynamic analyses.

#### 3.3.2 Equilibrium isotherm analysis

**Fig. 9:**
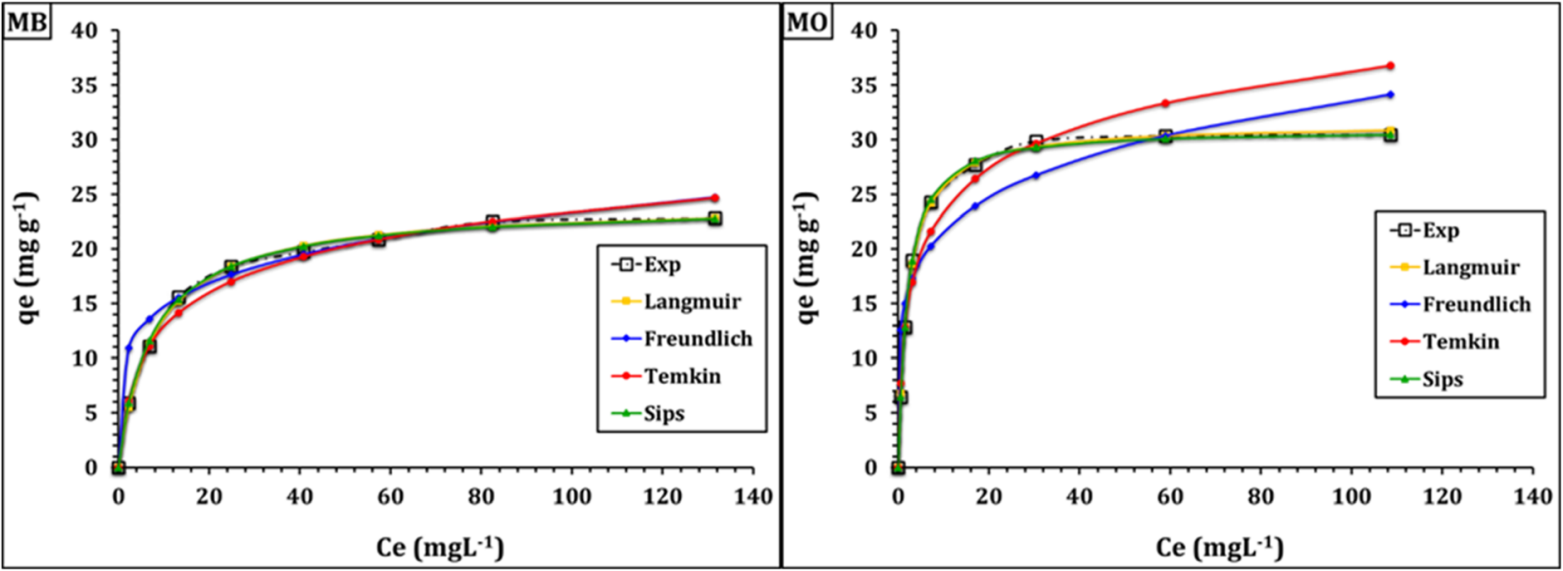
Isotherm profile for the adsorption of MB and MO species from aqueous using WRHB (temperature of 25 ± 1 °C; pH 6.01; sorbent dosage of 3.0 g L^−1^; time is 240 min).

The equilibrium adsorption behavior of methylene blue and methyl orange onto the synthesized WRHB composite was systematically evaluated using the Langmuir, Freundlich, Temkin, and Sips isotherm models. Equilibrium experiments were conducted at room temperature with initial dye concentrations ranging from 20 to 200 mg L^−1^, a sorbent dosage of 3.0 g L^−1^, and a contact time of 240 min to ensure complete equilibration. The experimental adsorption isotherms are shown in Fig. 9, and the corresponding model parameters and statistical indicators are summarized in Table 3.

**Table 3:** The values of the applied isotherm model parameters

	MB	MO
**Langmuir model**		
*q*_m_ (mg g^−1^)	24.2	31.5
*k*_L_ (L mg^−1^)	0.128	0.459
*R* ^2^	0.99	0.99
*X* ^2^	0.07	0.04

**Freundlich model**		
1/*n*_F_	0.20	0.19
*k*_F_ (mg g^−1^) (mg L^−1^)	9.2	13.9
*R* ^2^	0.92	0.83
*X* ^2^	2.9	5.6

**Temkin model**		
*b*_T_ (J mol^−1^)	540.2	442.8
*A*_T_ (L g^−1^)	1.6	6.6
*R* ^2^	0.97	0.92
*X* ^2^	0.4	2.2

**Sips model**		
*q*_S_ (mg g^−1^)	24.2	30.9
*k*_S_ (L mg^−1^)	0.13	0.48
*m*_S_	0.96	1.08
*R* ^2^	0.99	0.99
*X* ^2^	0.05	0.02

The Langmuir model provided an excellent representation of the equilibrium data for both dyes, as reflected by high correlation coefficients (*R*^2^ = 0.99 for MB and MO) and low chi-square values (*χ*^2^ = 0.07 for MB and 0.04 for MO). This strong agreement indicates that adsorption predominantly occurs through monolayer coverage on a finite population of energetically comparable surface sites.^41–43^ Such behavior is fully consistent with the relatively uniform dispersion of tungstate-derived inorganic domains identified by SEM-EDS and the presence of defined oxygen- and nitrogen-containing surface functionalities revealed by FTIR analysis. The calculated maximum monolayer adsorption capacities (*q*_m_) were 24.2 mg g^−1^ for MB and 31.5 mg g^−1^ for MO, clearly demonstrating the superior uptake capacity of the sorbent toward the anionic dye. This selectivity is further supported by the Langmuir affinity constants (*k*_L_), which were 0.128 L mg^−1^ for MB and 0.459 L mg^−1^ for MO, indicating stronger sorbate–sorbent interactions in the case of MO. The favorability of adsorption was further assessed using the dimensionless separation factor (*R*_L_ = 1/(1 + *k*_L_*C*_o_)). The calculated *R*_L_ values (Fig. S2) ranged from 0.038–0.281 for MB and 0.011–0.098 for MO across the investigated concentration range, confirming favorable adsorption behavior for both dyes (0 < *R*_L_ < 1).^41–43^ The consistently lower *R*_L_ values obtained for MO corroborate its stronger affinity and more favorable equilibrium behavior relative to MB, in agreement with the corresponding *k*_L_ and *q*_m_ parameters.

In contrast, the Freundlich model yielded comparatively weaker fitting performance, particularly for MO. The correlation coefficients decreased to 0.92 for MB and 0.83 for MO, accompanied by higher chi-square values (2.9 and 5.6, respectively). Although the Freundlich constants (*k*_F_) and intensity parameters (1/*n*_F_ = 0.19–0.20) indicate favorable adsorption, the reduced statistical reliability suggests that a purely heterogeneous multilayer adsorption description does not adequately represent the equilibrium data over the entire concentration range.^41–43^ This outcome aligns with the kinetic results, which indicated a multi-step adsorption process rather than unrestricted multilayer accumulation. The Temkin model exhibited intermediate fitting quality, with correlation coefficients of 0.97 for MB and 0.92 for MO and chi-square values of 0.4 and 2.2, respectively. The Temkin heat constants (*b*_T_) of 540.2 J mol^−1^ for MB and 442.8 J mol^−1^ for MO are relatively low, suggesting weak to moderate adsorption energies characteristic of predominantly physical interactions.^41–43^ These values imply that electrostatic attraction, π–π interactions, hydrogen bonding, and van der Waals forces contribute more significantly to adsorption than strong covalent bonding. The higher Temkin equilibrium constant (*A*_T_) for MO further confirms its stronger affinity toward the sorbent surface. Among the evaluated models, the Sips isotherm provided the most comprehensive description of the equilibrium adsorption behavior. The Sips model yielded maximum adsorption capacities (*q*_S_) of 24.2 mg g^−1^ for MB and 30.9 mg g^−1^ for MO, closely matching the Langmuir-derived *q*_m_ values. The affinity constants (*k*_S_) and heterogeneity parameters (*m*_S_ = 0.96 for MB and 1.08 for MO) indicate that, although minor surface heterogeneity exists, the adsorption process approaches Langmuir-type monolayer behavior, particularly at higher concentrations. The excellent fitting statistics (*R*^2^ = 0.99) and the lowest chi-square values among all tested models (0.05 for MB and 0.02 for MO) confirm that monolayer adsorption on energetically comparable sites dominates the equilibrium process.^41–43^

The equilibrium trends observed for WRHB are consistent with previously reported biomass-derived and hybrid sorbents designed for simultaneous removal of cationic and anionic dyes. Activated biocarbon derived from birchwood pellets and tamarind seed-based activated carbon systems demonstrated Langmuir-dominant behavior for MB and MO adsorption under comparable concentration conditions.^2,3^ Activated carbons prepared from mixed agricultural residues exhibited similar monolayer adsorption accompanied by concentration-dependent saturation.^14^ Nitrogen-functionalized carbon adsorbents have also been widely reported to follow Langmuir or Sips-type equilibrium behavior, as summarized in reviews on N-doped biochars and demonstrated experimentally for bagasse-derived N-doped biochar and nitrogen-rich seaweed carbon capable of dual dye removal.^24,26,28^ Hybrid inorganic–carbon systems, including WO_3_@TiO_2_/biochar heterojunctions, Mo–Bi_2_WO_6_/WO_3_/biochar composites, and related WO_3_-based biomass composites, likewise exhibited Langmuir-type equilibrium characteristics with heterogeneity parameters approaching unity.^34–37^ Rice husk-derived magnetic biochar used for dual dye adsorption also demonstrated favorable Langmuir behavior and enhanced affinity toward anionic species.^38^ These consistent findings confirm that the equilibrium performance of WRHB aligns mechanistically with established activated and inorganic-modified biochar platforms.

The consistently higher adsorption capacity and affinity toward MO compared with MB can be attributed to the combined influence of surface charge, chemical functionality, and inorganic domain incorporation. The pH_pzc_ value of approximately 8.6 indicates that, under the experimental conditions (pH ≈ 6), the sorbent surface is predominantly positively charged, thereby favoring electrostatic attraction toward the anionic sulfonate groups of MO while limiting electrostatic promotion of cationic MB adsorption.^17,26^ The presence of sodium tungstate oxide domains introduces polar and coordinatively active surface sites that further enhance interactions with anionic dye species.^34,38^ Although MB can associate with the aromatic carbon framework through π–π stacking and hydrogen bonding, these mechanisms are comparatively less selective under the prevailing charge conditions. Comparable preferential adsorption of MO over MB has been reported for activated biocarbon systems,^2^ Fe_3_O_4_/graphene oxide composites,^52^ wheat-straw-derived biochar,^53^ montmorillonite–chitosan gels,^54^ and magnetic mesoporous carbon materials,^55^ all of which attribute enhanced MO uptake to favorable electrostatic and polar surface interactions. Collectively, these parallels reinforce that the enhanced MO adsorption observed for WRHB is consistent with well-established charge-regulated adsorption behavior in modified biochar-based sorbents.

Overall, the equilibrium isotherm analysis demonstrates that adsorption of both MB and MO onto WRHB is best described by Langmuir- and Sips-type models, with MO consistently exhibiting higher adsorption capacity, affinity constants, and favorability parameters. The agreement between equilibrium modeling, kinetic interpretation, and physicochemical characterization provides a coherent and quantitatively supported description of the adsorption process and establishes a solid foundation for the subsequent thermodynamic evaluation.

#### 3.3.3 Thermodynamic analysis

**Fig. 10:**
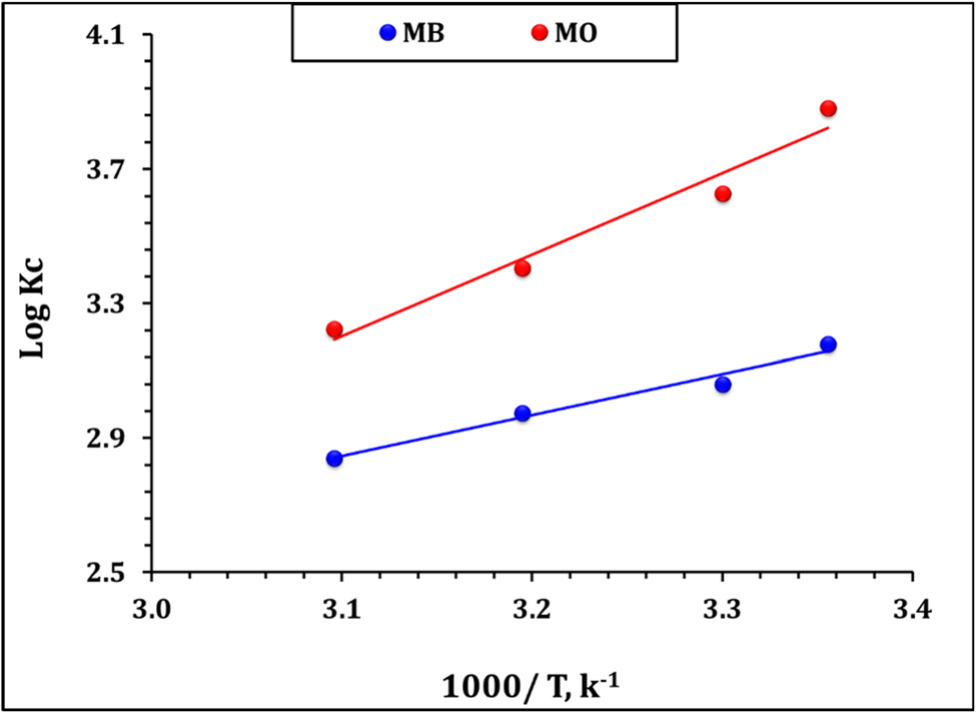
Thermodynamic profile for MB and MO adsorption process (40 mg L^−1^ initial concentration; sorbent dose of 3.0 g L^−1^; pH 5.9; shaking time is 120 min).

The thermodynamic feasibility and intrinsic nature of methylene blue (MB) and methyl orange (MO) adsorption onto the WRHB composite were evaluated through determination of the standard Gibbs free energy change (Δ*G*°), enthalpy change (Δ*H*°), and entropy change (Δ*S*°). These parameters were derived from temperature-dependent adsorption data collected over the range of 25–50 °C using the Van't Hoff relationship (Fig. 10), and the calculated values are summarized in Table 4.

**Table 4:** The evaluated thermodynamic parameters

	Δ*G* (kJ mol^−1^)				Δ*H* (kJ mol^−1^)	Δ*S* (J mol^−1^ K^−1^)
	25 °C	30 °C	40 °C	50 °C		
MB	−18.1	−17.8	−17.8	−17.6	−23.4	−17.6
MO	−22.1	−21.0	−20.4	−19.9	−46.3	−81.2

The Δ*G*° values for both dyes were negative throughout the investigated temperature range, confirming the spontaneous nature of the adsorption process.^44,45^ For MB, Δ*G*° values decreased slightly in magnitude from −18.1 kJ mol^−1^ at 25 °C to −17.6 kJ mol^−1^ at 50 °C, while for MO, the values ranged from −22.1 to −19.9 kJ mol^−1^ over the same interval. The more negative Δ*G*° values obtained for MO indicate a stronger thermodynamic driving force for MO adsorption compared with MB, in full agreement with the kinetic and equilibrium analyses that demonstrated higher affinity constants and faster uptake of the anionic dye. The gradual reduction in the magnitude of Δ*G*° with increasing temperature indicates that adsorption becomes less favorable at elevated temperatures, consistent with the experimentally observed decline in removal efficiency and adsorption capacity under higher thermal conditions.^26,44,45^

The calculated Δ*H*° values were −23.4 kJ mol^−1^ for MB and −46.3 kJ mol^−1^ for MO, confirming that adsorption of both dyes is exothermic.^40,41^ The more negative enthalpy change for MO reflects stronger dye–surface interactions relative to MB, further corroborating the preferential adsorption behavior established in the isotherm analysis. Importantly, the magnitude of Δ*H*° for both dyes falls within the range commonly associated with physical adsorption and weak specific interactions rather than strong covalent bonding.^44,45^ This energetic range is characteristic of adsorption governed by electrostatic attraction, hydrogen bonding, π–π interactions, and surface complexation involving polar oxide domains, as widely reported for modified biochar and inorganic-functionalized carbon composites.^38,50,51,56,57^ The enthalpy values therefore support a predominantly physisorption-driven mechanism enhanced by specific but non-covalent surface interactions. The entropy change (Δ*S*°) values were negative for both dyes, with −17.6 J mol^−1^ K^−1^ for MB and −81.2 J mol^−1^ K^−1^ for MO. Negative Δ*S*° values indicate a decrease in randomness at the solid–liquid interface during adsorption, reflecting the structured arrangement of dye molecules onto defined active sites and the restriction of molecular mobility upon surface attachment.^44,45^ The more negative entropy change observed for MO suggests a higher degree of ordering at the interface, consistent with its stronger affinity and greater surface coverage inferred from Langmuir and Sips modeling results.

Taken together, the thermodynamic parameters confirm that adsorption of MB and MO onto WRHB is spontaneous and exothermic, with MO adsorption being thermodynamically more favorable. The close agreement between Δ*G*°, Δ*H*°, and Δ*S*° trends and the experimentally observed temperature-dependent adsorption behavior demonstrates the internal coherence of the system. Furthermore, the moderate magnitudes of Δ*H*° and the negative entropy changes are consistent with the surface chemistry characteristics established through physicochemical characterization, particularly the presence of positively charged sites and tungstate-derived polar domains that promote structured interfacial interactions. Comparable thermodynamic profiles—marked by negative Δ*G*°, exothermic Δ*H*°, and reduced interfacial entropy—have been documented for rice husk-derived magnetic biochar employed in dual dye adsorption systems,^38^ activated carbon/polymer nanocomposite adsorbents for methylene blue removal,^50^ N/O co-doped biomass porous carbon exhibiting strong affinity toward methyl orange,^51^ and carboxymethyl cellulose-grafted magnetic biochar hydrogels designed for efficient cationic dye elimination.^56,57^ The recurrence of these thermodynamic signatures across structurally diverse sorbent platforms highlights a common adsorption pathway governed predominantly by physical interactions and surface-regulated affinity mechanisms. Overall, the thermodynamic evaluation provides quantitative confirmation of the selective adsorption behavior of WRHB and substantiates its operational suitability under ambient temperature conditions, thereby reinforcing its potential applicability in practical wastewater treatment scenarios.

### 3.4 The proposed mechanism

Clarifying the adsorption pathways operating at the solid–liquid interface is essential for interpreting the observed dye uptake behavior and for guiding the rational design of high-performance sorbents for wastewater treatment. In biochar- and carbon-composite-based systems, adsorption is rarely governed by a single dominant interaction; rather, overall uptake generally arises from the cooperative contribution of multiple mechanisms, including electrostatic attraction or repulsion, hydrogen bonding, π–π interactions between aromatic structures, ion–dipole interactions, donor–acceptor interactions, surface complexation on inorganic domains, and physical adsorption *via* pore filling and van der Waals forces. The relative contribution of these pathways depends on the sorbent's physicochemical characteristics—particularly its surface charge behavior, distribution of functional groups, hybrid organic–inorganic architecture, and pore structure—as well as on the molecular features of the adsorbates, such as charge state, aromaticity, and functional substituents under specific solution conditions.^17,24–27,33–35,38^

In the present study, adsorption of methylene blue (MB) and methyl orange (MO) onto WRHB proceeds through a cooperative multi-interaction mechanism governed by the interplay between pH-dependent surface charge characteristics, tungstate-derived inorganic domains dispersed within the carbon framework, oxygen-containing surface functionalities identified by FTIR analysis, and the accessible mesoporosity revealed by textural measurements. The hybrid structure of WRHB—comprising amorphous carbon domains that provide π-electron-rich regions alongside crystalline sodium tungstate oxide domains that introduce polar and coordinatively active sites—creates a chemically heterogeneous adsorption interface capable of accommodating both cationic and anionic dyes through complementary interaction routes.

Electrostatic interactions provide the fundamental framework for understanding the observed selectivity. The experimentally determined pH_pzc_ value of approximately 8.6 indicates that, under the working conditions employed in this study (near-neutral pH and below pH_pzc_), the WRHB surface carries a net positive charge. This surface environment strongly promotes coulombic attraction toward the anionic sulfonate groups of MO, thereby facilitating efficient initial adsorption. In contrast, MB, being a cationic dye, does not benefit from electrostatic attraction under these conditions and therefore relies more heavily on alternative interaction pathways. This electrostatic interpretation is fully consistent with the stronger affinity constants, lower separation factors, and more negative Gibbs free energy values obtained for MO in the equilibrium and thermodynamic analyses.^17,25–27^ Beyond electrostatic considerations, the incorporation of crystalline sodium tungstate oxide domains, as confirmed by XRD and SEM-EDS analyses, introduces polar metal–oxygen-rich sites capable of participating in ion–dipole interactions and localized surface complexation with dye molecules.^33–35,38^ Such polar interactions are intrinsically more favorable for anionic species and therefore contribute to the enhanced stabilization of MO at the sorbent surface. Simultaneously, FTIR results revealed abundant oxygen-containing functional groups, including hydroxyl and metal–oxygen moieties, which further support hydrogen bonding and specific polar interactions. These combined effects establish a cooperative binding environment in which electrostatic anchoring, polar interactions, and hydrogen bonding collectively strengthen MO adsorption.

MB adsorption is governed primarily by interactions with the carbonaceous framework. Its extended aromatic structure facilitates π–π stacking with the conjugated domains of the biochar matrix, while hydrogen bonding with oxygen- and nitrogen-containing surface functionalities and confinement within accessible mesopores provide supplementary stabilization. These non-electrostatic interactions enable substantial MB uptake despite the less favorable electrostatic environment. The lower affinity of MB relative to MO therefore reflects differences in the relative contributions of the available interaction pathways rather than the operation of fundamentally different adsorption regimes.

**Fig. 11:**
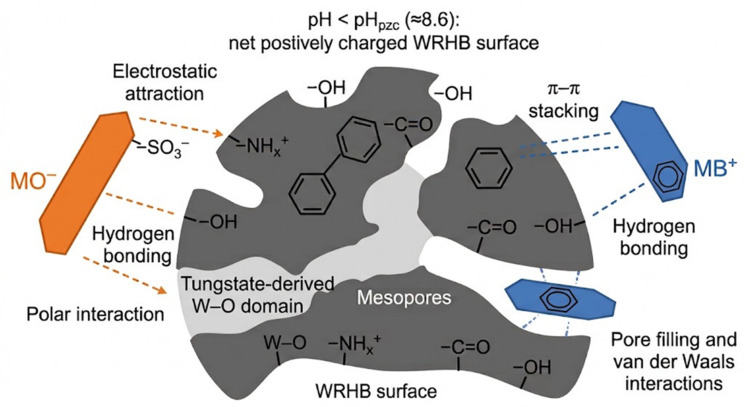
Simplified schematic representation of the proposed interactions governing MO and MB adsorption onto WRHB. Fully generated with Google Nano Banana and scientifically reviewed and verified by the authors.

The kinetic and thermodynamic results further reinforce this mechanistic interpretation. The excellent conformity of both dyes to the pseudo-second-order model indicates that adsorption is governed by the progressive occupation of available surface sites and by the strength of dye–surface interactions rather than by simple first-order diffusion processes. The non-zero intercepts obtained from the Weber–Morris intraparticle diffusion analysis demonstrate that intraparticle diffusion is not the sole rate-controlling step and that boundary-layer mass transfer contributes to the overall uptake mechanism, which is characteristic of heterogeneous porous sorbents where multiple transport regimes operate sequentially. Thermodynamically, the negative Δ*G*° values confirm spontaneous adsorption, while the negative Δ*H*° values indicate an exothermic process consistent with temperature-dependent experimental trends. The moderate enthalpy magnitudes fall within the range typically associated with physical adsorption and weak specific interactions rather than strong covalent bonding, and the negative Δ*S*° values reflect a reduction in interfacial randomness resulting from the ordered arrangement of dye molecules upon adsorption. Notably, the more favorable thermodynamic parameters obtained for MO coherently align with the stronger electrostatic and polar-site contributions described above, providing an energetically consistent explanation for the preferential adsorption of the anionic dye. This interpretation is consistent with previous studies of carbon-based and hybrid sorbents, in which aromatic-dye adsorption has been attributed to cooperative electrostatic, π–π, hydrogen-bonding, polar-site, and pore-filling interactions. Tungsten- and tungstate-modified carbon materials additionally provide metal–oxygen-rich domains that can enhance polar interactions with adsorbate molecules.^24,26,27,33–35,38,46,56^ The proposed interaction pathways governing MB and MO adsorption onto WRHB are summarized schematically in Fig. 11.

**Fig. 12:**
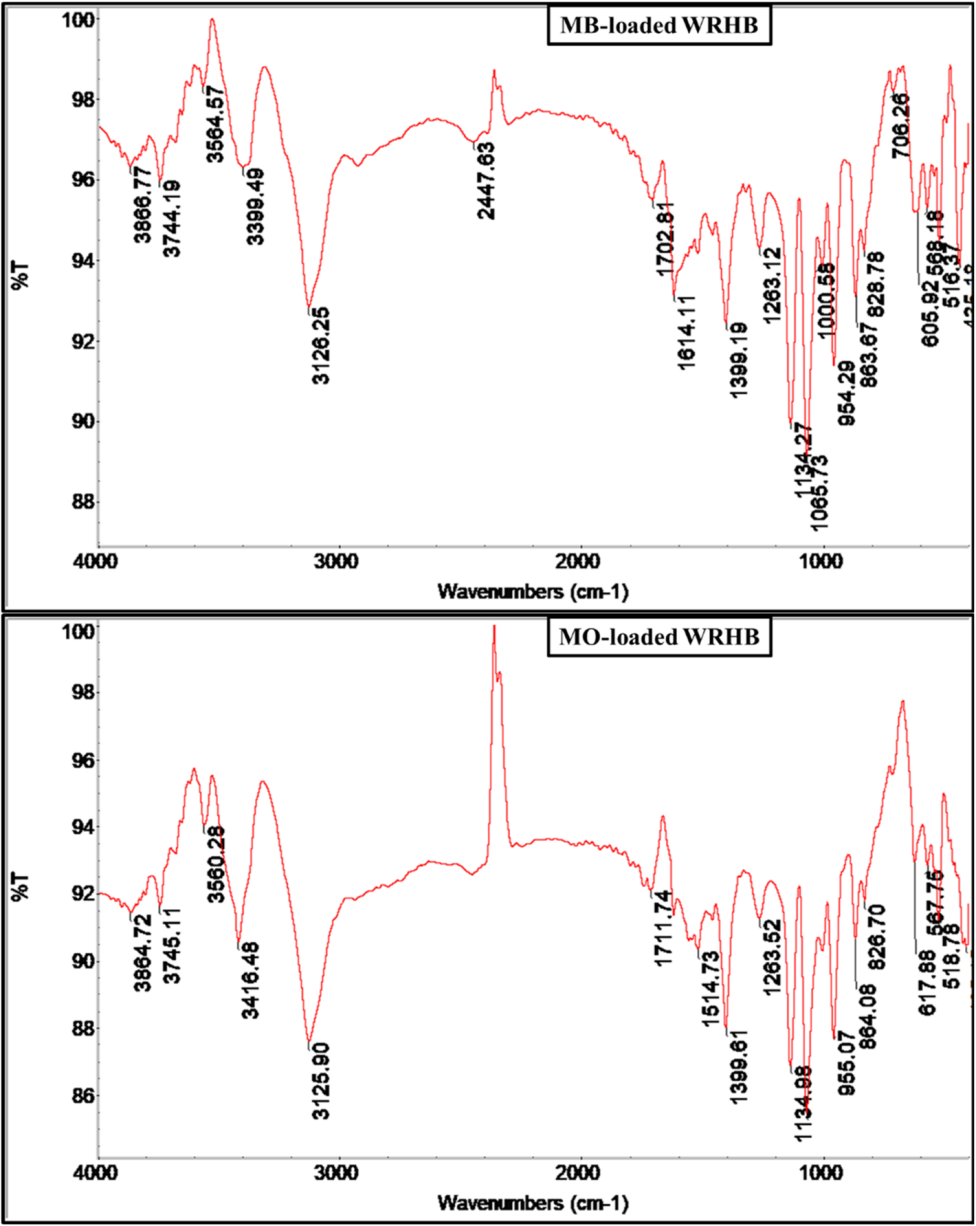
FTIR spectra of (a) MB-loaded WRHB and (b) MO-loaded WRHB after adsorption.

Further experimental support for the proposed interaction pathways was obtained by comparing the FTIR spectra of MB-loaded and MO-loaded WRHB (Fig. 12) with the pristine WRHB spectrum shown in Fig. 2. The broad O–H/N–H envelope centered at 3271.47 cm^−1^ in pristine WRHB exhibited broad minima at 3126.25 cm^−1^ for MB-loaded WRHB and 3125.90 cm^−1^ for MO-loaded WRHB. These profile changes indicate perturbation of the hydroxyl- and nitrogen-containing surface environments and are consistent with their participation in hydrogen-bonding and polar interactions. Because this region contains overlapping vibrations, the observed changes are not assigned to a single functional group or bonding pathway. The pristine band at 1446.09 cm^−1^ was no longer resolved as an isolated feature after adsorption, while prominent bands appeared at 1399.19 cm^−1^ for MB-loaded WRHB and 1399.61 cm^−1^ for MO-loaded WRHB. The tungstate-related W–O band also moved from 837.31 cm^−1^ in pristine WRHB to 828.78 cm^−1^ after MB adsorption and 826.70 cm^−1^ after MO adsorption, supporting perturbation of the tungstate-derived polar domains during dye uptake. Additional bands were observed at 1702.81, 1614.11, 1263.12, 1134.27, 1065.73, and 954.29 cm^−1^ for MB-loaded WRHB and at 1711.74, 1514.73, 1263.52, 1134.98, and 955.07 cm^−1^ for MO-loaded WRHB. These fingerprint-region features confirm the presence of retained dye molecules on the sorbent and demonstrate modification of the WRHB vibrational environment following adsorption. The broadly similar spectral responses indicate that MB and MO interact with a common set of surface functionalities, whereas the distinct bands at 1614.11 cm^−1^ for MB-loaded WRHB and 1514.73 cm^−1^ for MO-loaded WRHB reflect dye-specific spectral contributions. Because absolute FTIR intensities are sensitive to sample preparation and contact conditions, the interpretation is based on band positions and spectral profiles rather than intensity differences. Accordingly, the FTIR observations support the participation of surface functionalities and tungstate-derived domains but are not interpreted as evidence of covalent bond formation.

Overall, MO adsorption is favored by electrostatic attraction to the positively charged WRHB surface, together with hydrogen bonding and interactions with tungstate-derived polar sites. MB adsorption depends more strongly on π–π stacking, hydrogen bonding, and pore filling within the carbonaceous mesoporous framework. The combined evidence from pH-dependent adsorption, equilibrium and thermodynamic parameters, kinetic modeling, and post-adsorption FTIR analysis therefore supports a cooperative multi-pathway mechanism and explains the preferential affinity of WRHB toward MO.

### 3.5 Desorption, regeneration, and practical application assessment

From a practical application standpoint, the ability to regenerate an adsorbent using simple, low-cost, and readily available reagents is a critical criterion for its feasibility in real wastewater treatment systems. To evaluate the reusability potential of WRHB, desorption experiments were conducted using 1.0 M HCl, H_2_SO_4_, and HNO_3_, and the resulting desorption efficiencies for methylene blue (MB) and methyl orange (MO) are depicted in Fig. S3. The obtained results clearly demonstrate that WRHB exhibits high regeneration efficiency, with a pronounced dependence on the type of eluting agent employed. Among the tested eluents, 1.0 M HCl showed the highest desorption performance, achieving desorption efficiencies of 87.5% for MB and 94.8% for MO. In comparison, H_2_SO_4_ resulted in moderate desorption efficiencies (68.9% for MB and 76.1% for MO), while HNO_3_ exhibited comparatively lower desorption performance (52.6% and 58.6%, respectively). These results highlight a key practical advantage of the WRHB sorbent: effective regeneration can be accomplished using a single, inexpensive, and widely available mineral acid without reliance on organic solvents, complex chemical eluents, or energy-intensive thermal treatments. Such regeneration behavior is particularly attractive for large-scale and continuous wastewater treatment operations, where operational simplicity, chemical availability, and cost-effectiveness are decisive factors for industrial implementation.

To evaluate WRHB performance under realistic conditions, the sorbent was applied to a complex wastewater matrix containing multiple dissolved constituents and a measurable color load. The initial composition and the corresponding removal efficiencies are summarized in Table S5. Under these realistic conditions, WRHB achieved a high color-intensity removal of 85.4%, demonstrating that the sorbent retains strong decolorization capability even in the presence of competing ions and dissolved species. While total dissolved solids (TDS) were only partially reduced (35.9%), WRHB exhibited different removal efficiencies toward the measured metal ions, reaching 59.4% for Zn, 44.4% for Cd, 38.1% for Mn, and 32.9% for Al, whereas lower removal efficiencies were observed for Cu (24.1%) and Fe (23.4%). Notably, color-intensity removal was considerably higher than the removal of TDS and all measured metal ions, indicating preferential removal of the chromophoric organic constituents responsible for wastewater coloration, together with supplementary uptake of selected inorganic species. This matrix-level preference is relevant to textile-wastewater treatment, where color reduction represents a central treatment objective. Importantly, the 85.4% decolorization achieved in the real wastewater remained substantial despite competition from coexisting organic and inorganic constituents. This result indicates that WRHB retains its adsorption function under complex matrix conditions. Because the color-intensity measurement represents the combined optical contribution of the wastewater colorants, the individual removal efficiencies and competitive selectivities of MB and MO cannot be independently distinguished in this matrix. Nevertheless, the substantially greater reduction in color intensity than in the measured inorganic constituents supports preferential decolorization at the matrix level rather than strict solute-specific selectivity.

Overall, the combined desorption and application results demonstrate that WRHB combines high desorption efficiency, effective regeneration, operational simplicity, and sustained decolorization performance in a real wastewater matrix. The ability to achieve efficient dye desorption using simple mineral acids, together with sustained decolorization efficiency under complex conditions, supports the practical relevance of WRHB as an application-oriented adsorbent. When coupled with its straightforward preparation from waste biomass and stable adsorption behavior, these advantages underscore the potential of WRHB for the treatment of dye-containing wastewater.

## 4 Conclusion

This study demonstrates that rational integration of alkali activation, nitrogen functionalization, and tungstate-derived inorganic domains within a rice husk biochar matrix produces a chemically heterogeneous yet structurally stable hybrid sorbent capable of efficient dual-dye adsorption. Comprehensive physicochemical characterization confirmed the formation of an amorphous carbon framework decorated with crystalline Na_2_WO_4_ domains, moderate mesoporosity, accessible surface area, and an alkaline pH_pzc_ conducive to charge-regulated adsorption. Batch adsorption experiments established that dye removal is governed by the interplay between surface charge behavior, pore accessibility, and chemical functionality. Methyl orange consistently exhibited higher adsorption capacity and affinity than methylene blue, reflecting the favorable electrostatic and polar-site interactions supported by tungstate domains and oxygenated functionalities. Kinetic modeling confirmed pseudo-second-order behavior with multi-step diffusion contributions, while equilibrium analysis demonstrated Langmuir–Sips dominance, indicating primarily monolayer adsorption on energetically comparable sites. Thermodynamic parameters verified spontaneous and exothermic adsorption governed predominantly by physical and weak specific interactions. Importantly, WRHB exhibited high regeneration efficiency using simple mineral acids and maintained strong performance in real textile wastewater, confirming structural robustness and operational practicality. The combination of scalable biomass-derived synthesis, multifunctional adsorption pathways, selective affinity toward anionic dyes, and effective real-matrix performance highlights the practical relevance of this hybrid sorbent design. Overall, this work advances rice husk-derived biochar composites from single-function laboratory materials toward application-oriented multifunctional platforms suitable for real wastewater treatment systems.

## Conflicts of interest

The authors declare that they have no known financial or non-financial interests, direct or indirect, related to the submitted work within the past three years or beyond, that could reasonably be perceived as influencing the manuscript.

## Supplementary Material

RA-OLF-D6RA02755E-s001

## Data Availability

Supplementary information (SI): adsorption-model equations, Weber–Morris parameters and plots, dimensionless separation-factor data, desorption results, dye physicochemical properties, and the initial and final characteristics of the real textile wastewater. Additional datasets are available from the corresponding author upon reasonable request. See DOI: https://doi.org/10.1039/d6ra02755e.
