# Supplementary material for: Hybrid tungstate-functionalized nitrogen-doped rice husk biochar for selective dual adsorption of methylene blue and methyl orange
Source: RSC Adv. 2026 Aug 5;16(39):44998–5017. doi: 10.1039/d6ra02755e (PMC13440589; doi:10.1039/d6ra02755e)
Supplement: Supplementary file 1 [file RA-016-D6RA02755E-s001.pdf]

Supplementary Information (SI) for RSC Advances.
This journal is © The Royal Society of Chemistry 2026

Supplementary Information

Hybrid Tungstate-Functionalized Nitrogen-Doped Rice Husk Biochar for Selective Dual Adsorption of Methylene Blue and Methyl Orange

Walid M. Youssef [**1**]***, Entsar H. Taha***[**2**]***, Adel A. El-Zahhar***[**3**]***, Majed M. Alghamdi***[**3**]***, Mohamed H. Taha***[**1**]

[1] Nuclear Materials Authority, P.O. Box 530, El Maddi, Cairo, Egypt

[2]Department of Plant Protection, Faculty of Agriculture, Ain Shams University, Cairo, Egypt

[3]Department of Chemistry, Faculty of Science, King Khalid University, P.O.Box 9004, Abha 61413, Saudi Arabia

* Corresponding author: m.walid_nma@yahoo.com

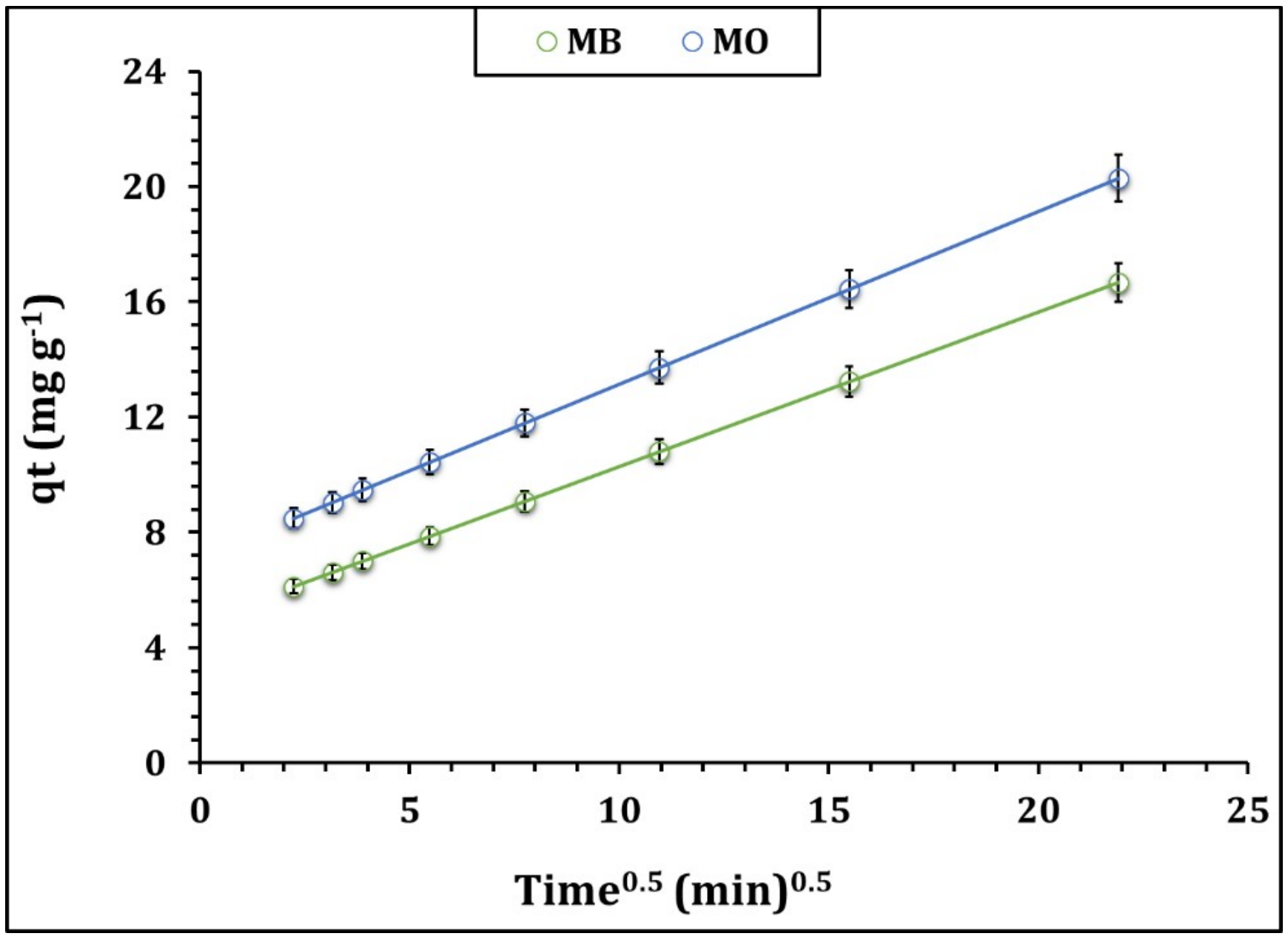

Figure S1: *W-M model illustrate for uptake process (solution pH 6.02; WRHB dosage of 3.0 g L^{-1}; initial concentration of 50 mg/ L; temperature of 25±1°C).*

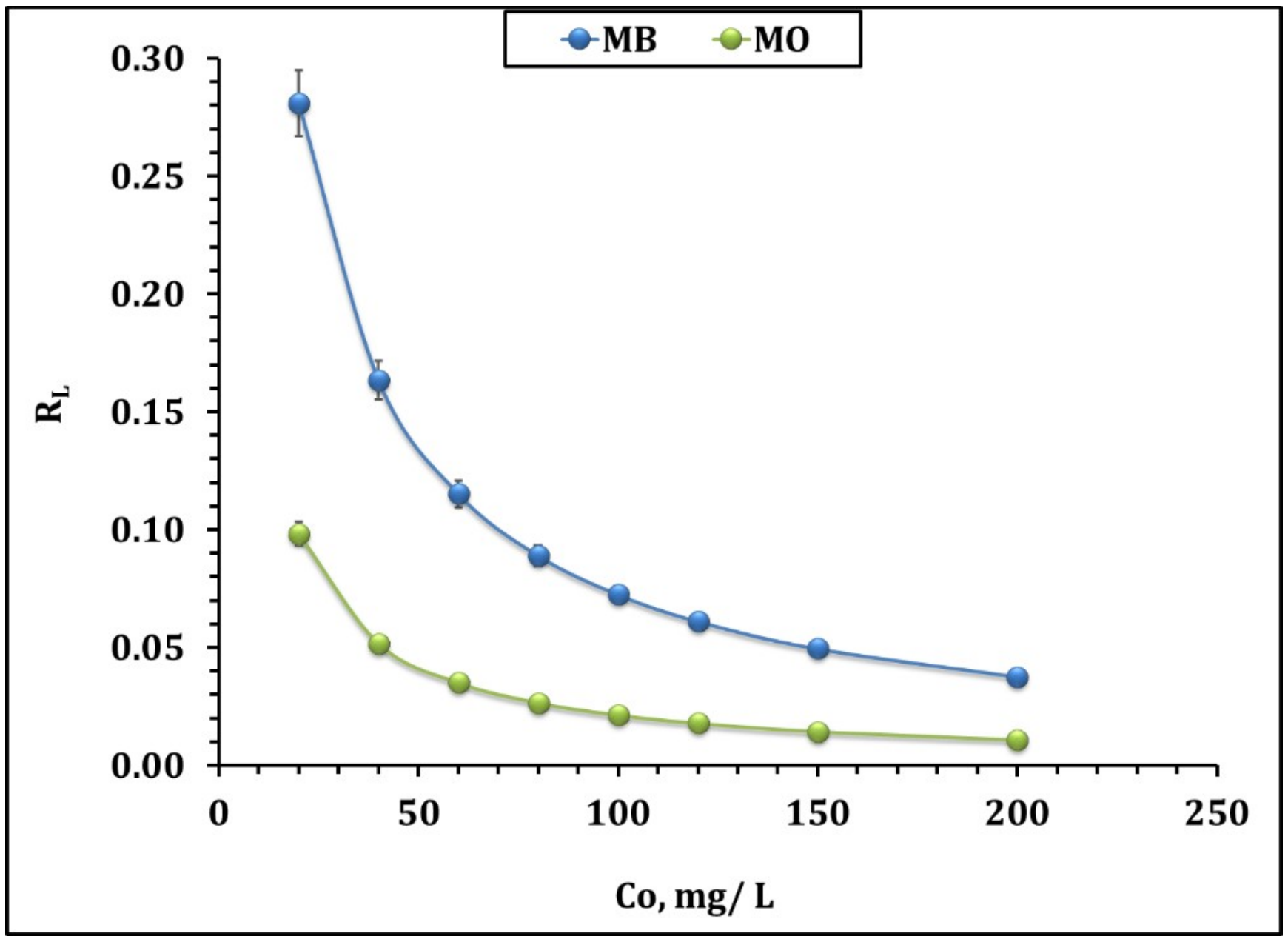

Figure S2: *Dimensionless equilibrium parameter* R_L *for the adsorption of MB and MO species.*

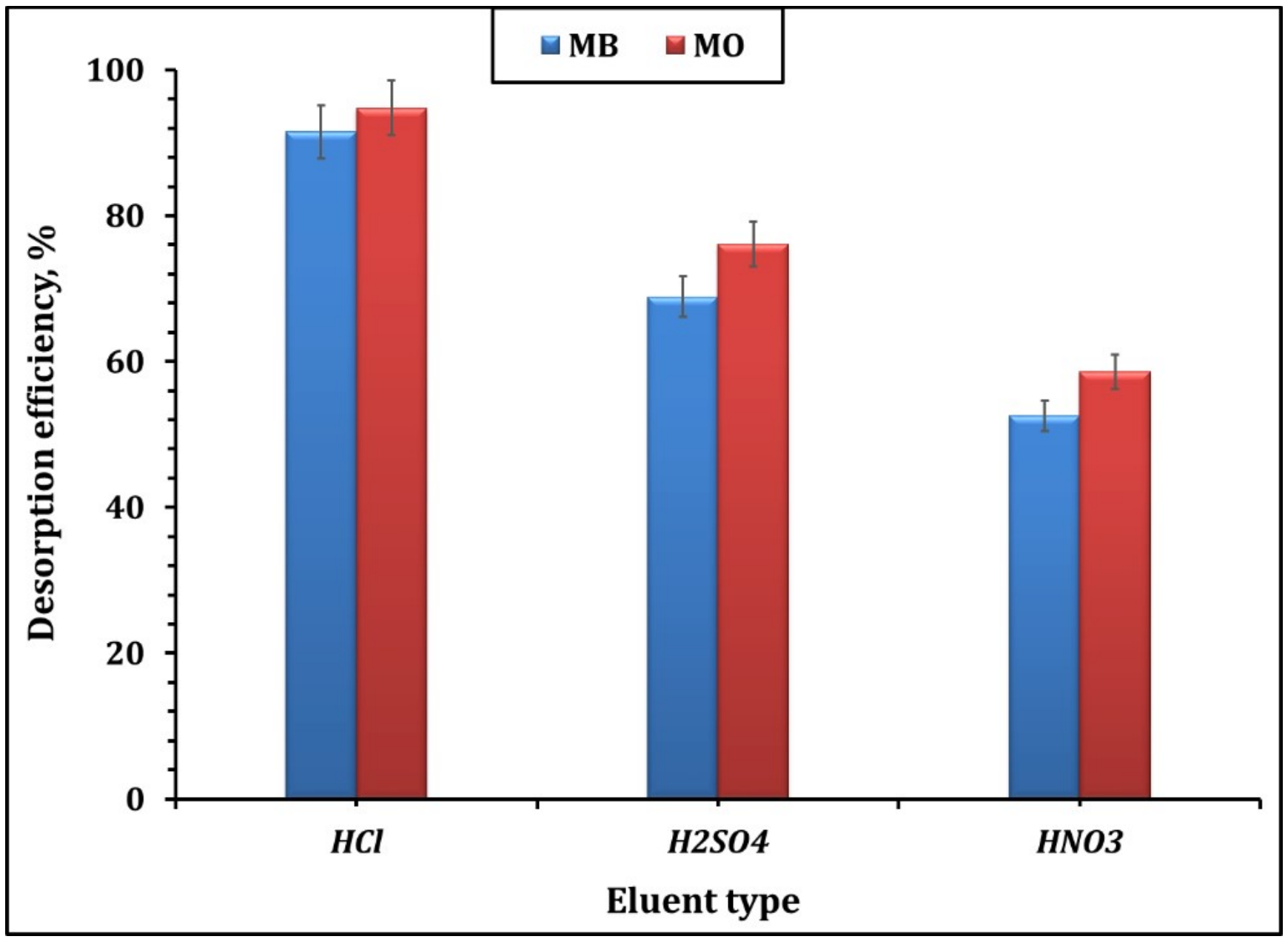

Figure S3: *MB and MO desorption from loaded sorbent using different solutions (3.0 g/L, room temperature; 240 min).*

Table S1: *Physicochemical properties of Methyl orange and crystal violet.*

Dye	Methyl orange	Methylene blue
IUPAC name	Sodium 4-[(4-dimethylamino)phenylazo]benzenesulfonate	3,7-bis(Dimethylamino)phenothiazin-5-ium chloride
Type	Anionic dye	Cationic dye
Molecular weight	327.33 g·mol^{-1}	319.85 g·mol^{-1}
λ max	464 nm	664 nm
Molecular formula	$C_{14}H_{14}N_3NaO_3S$	$C_{16}H_{18}ClN_3S$
Appearance	Orange or yellow solid	Dark blue
Structure	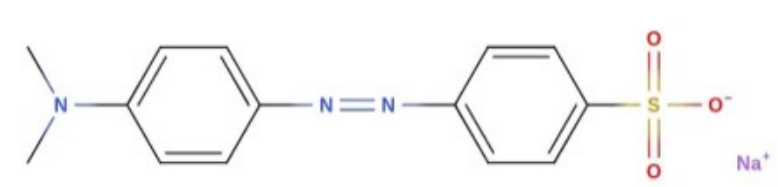	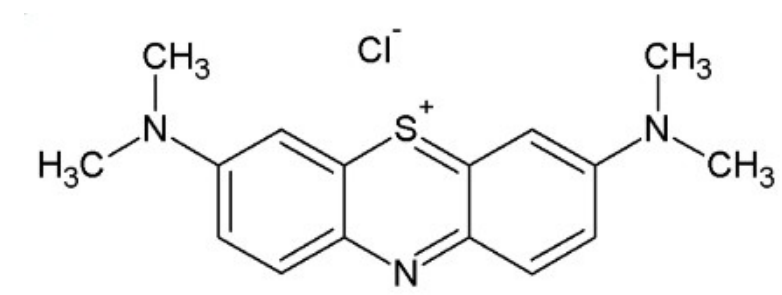

Table S2: *Initial concentrations of constituents in raffinate wastewater.*

**Parameter**	**Initial concentration, mg/ L**
Cu(II)	9.0
Cd(II)	0.5
Fe(III)	29.0
T.D.S	270.0
Al(III)	9.0
Mn(II)	11.8
Zn(II)	9.0
Color intensity	10.0

Table S3: *Kinetic, isotherm, and thermodynamics equations for MO and MB, sorption process* ***[1-7]***.

Kinetics	Equations
**Pseudo-first-order**	$q_t = q_1\left(1 - e^{-k1t}\right)$
**Pseudo-second-order**	$q_t = \frac{1}{\left(1 \mid k_2 q_2^2\right) + \left(t \mid q_2\right)}$
**Intra-particle diffusion model (IPD)**	$q_t = K_{id}\, t^{0.5} + C_i$
**Isotherms**	**Equations**
**Langmuir model**	$q_e = \frac{q_m k_L C_e}{1 + k_L C_e}$
**Freundlich model**	$q_e = K_F C_e^{1/n_F}$
**Temkin model**	$q_e = \frac{RT}{b_T} \ln K_T C_e$
**Sips model**	$q_e = \frac{q_S \left(k_S C_e\right)^{mS}}{1 + \left(k_S C_e\right)^{mS}}$
**Thermodynamics**	**Equations**
$\log K_C = -\frac{\Delta H^o}{2.303\, R} X \frac{1}{T} + C$	
$-\Delta G^o = 2.303\, RT \log K_C$	
$\Delta G^o = \Delta H^o - T\, \Delta S^o$	
**Fitting**	**Equations**
**Coordination coefficient (R^2)**	$R^2 = 1 - \frac{\sum_1^n \left(q_{exp} - q_{pred}\right)^2}{\sum_1^n \left(q_{exp} - \bar{q}_{exp}\right)^2}$
**Chi-square coefficient (x^2)**	$x^2 = \sum \left[\frac{\left(q_{exp} - q_{pred}\right)^2}{q_{pred}}\right]$

qe (mg g^{-1}) is the equilibrium concentration of MB species, and qt (mg g^{-1}) is the adsorbed amount of dyes species ions after time t (min), Ce (mg L^{-1}) is equilibrium concentration of dyes species. k_1 (min^{-1}) and k_2 (min^{-1}) are the rate constants for the pseudo first and second order, respectively. K_{id} (mg/g. $min^{0.5}$) is a rate constant, and C is the thickness of the boundary layer. q_m and q_S are the maximum sorption capacity (mg. g^{-1}) of Langmuir and Sips models. k_L (L. mg^{-1}), K_F (L/ mg), K_T (L min^{-1}), and K_S (L/ mg) are represent the constants of Langmuir, Freundlich, Temkin, and Sips models. n refer to the sorption intensity, b_T is Temkin constant that refers to the adsorption heat, mS is Sips constant. $\boldsymbol{q_s}$ is the theoretical isotherm saturation capacity (mg/g). K_C is a non-dimensional equilibrium constant and it equals K_d X 1000 X ρ **[4-5]**; T is the temperature (K), $\boldsymbol{R}$ is the universal gas constant (8.314 J mol^{-1}. K^{-1}), ρ is solution density g/ L, and C is a constant. R^2 and x^2 are the coordination and Chi-square coefficients respectively, the number of test points is n, the experimental equilibrium capacity is q_{exp} (mg g^{-1}), while the predicted capacity is q_{pred} (mg g^{-1}).

References:

1. Q. Hu, S. Pang and D. Wang, *Sep. Purif. Rev.*, 2022, *51*, 281–299.
2. M. E. González-López, C. M. Laureano-Anzaldo, A. A. Pérez-Fonseca, M. Arellano and J. R. Robledo-Ortíz, *Sep. Purif. Rev.*, 2022, *51*, 358–372.
3. M. H. Taha, *Environ. Sci. Pollut. Res.*, 2021, *28*, 12475–12489.
4. X. Chen, M. F. Hossain, C. Duan, J. Lu, Y. F. Tsang, M. S. Islam and Y. Zhou, *Chemosphere*, 2022, *307*, 135545.
5. M. M. Majd, V. Kordzadeh-Kermani, V. Ghalandari, A. Askari and M. Sillanpää, *Sci. Total Environ.*, 2022, *812*, 151334.
6. A. M. Masoud, H. Ammar, A. A. Elzoghby, H. H. El Agamy and M. H. Taha, *J. Rare Earths*, 2025, *43*, 1502–1512.
7. A. N. Ebelegi, N. Ayawei and D. Wankasi, *Open J. Phys. Chem.*, 2020, *10*, 166–182.

Table S4: *The values of the Weber and Morris model parameters.*

		MB	MO
**Weber and Morris model**	**k_i (mg/g min$^{1/2}$)**	0.54	0.60
	**C**	4.9	7.1
	**R^2**	0.98	0.98

Table S5: *Initial and final characteristics of the real textile wastewater and corresponding removal efficiencies following treatment with WRHB.*

Parameter	Unit	Initial value	Final value	Removal efficiency (%)
Cu(II)	mg L^{-1}	9.0	6.8	24.1
Cd(II)	mg L^{-1}	0.5	0.3	44.4
Fe(III)	mg L^{-1}	29.0	22.2	23.4
TDS	mg L^{-1}	270.0	173.0	35.9
Al(III)	mg L^{-1}	9.0	6.0	32.9
Mn(II)	mg L^{-1}	11.8	7.3	38.1
Zn(II)	mg L^{-1}	9.0	3.7	59.4
Color intensity	Absorbance (a.u.)	10.0	1.5	85.4
